# Nuclear and Radiological Emergencies: Biological Effects, Countermeasures and Biodosimetry

**DOI:** 10.3390/antiox11061098

**Published:** 2022-05-31

**Authors:** Elena Obrador, Rosario Salvador-Palmer, Juan I. Villaescusa, Eduardo Gallego, Blanca Pellicer, José M. Estrela, Alegría Montoro

**Affiliations:** 1 Department of Physiology, Faculty of Medicine and Odontology, University of Valencia, 46010 Valencia, Spain; rosario.salvador@uv.es (R.S.-P.); b.pellicerdegracia@gmail.com (B.P.); jose.m.estrela@uv.es (J.M.E.); 2 Service of Radiological Protection, Clinical Area of Medical Image, La Fe University Hospital, 46026 Valencia, Spain; villaescusa_ign@gva.es (J.I.V.); almonpas@hotmail.com (A.M.); 3 Biomedical Imaging Research Group GIBI230, Health Research Institute (IISLaFe), La Fe University Hospital, 46026 Valencia, Spain; 4 Energy Engineering Department, School of Industrial Engineering, Polytechnic University of Madrid, 28040 Madrid, Spain; eduardo.gallego@upm.es

**Keywords:** nuclear and radiological emergencies, radioprotectors, radiomitigators, radionuclide scavengers, radiation biodosimetry

## Abstract

Atomic and radiological crises can be caused by accidents, military activities, terrorist assaults involving atomic installations, the explosion of nuclear devices, or the utilization of concealed radiation exposure devices. Direct damage is caused when radiation interacts directly with cellular components. Indirect effects are mainly caused by the generation of reactive oxygen species due to radiolysis of water molecules. Acute and persistent oxidative stress associates to radiation-induced biological damages. Biological impacts of atomic radiation exposure can be deterministic (in a period range a posteriori of the event and because of destructive tissue/organ harm) or stochastic (irregular, for example cell mutation related pathologies and heritable infections). Potential countermeasures according to a specific scenario require considering basic issues, e.g., the type of radiation, people directly affected and first responders, range of doses received and whether the exposure or contamination has affected the total body or is partial. This review focuses on available medical countermeasures (radioprotectors, radiomitigators, radionuclide scavengers), biodosimetry (biological and biophysical techniques that can be quantitatively correlated with the magnitude of the radiation dose received), and strategies to implement the response to an accidental radiation exposure. In the case of large-scale atomic or radiological events, the most ideal choice for triage, dose assessment and victim classification, is the utilization of global biodosimetry networks, in combination with the automation of strategies based on modular platforms.

## 1. Introduction

Nuclear and radiological accidents can cause huge harm to individuals, the environment, and the economy. Chernobyl (USSR, 1986), Goiania (Brazil, 1987), and Fukushima Daiichi (2011, Japan) were awful catastrophes demonstrating how wrecking these mishaps can be. Moreover, since 11 September 2001, the danger of terrorism has become a public security concern in numerous nations. The number of known terrorist associations with worldwide reach, just like the expanded multiplication and transfer of technical data through the web, raises the chance of shocking assaults with chemical, biological, radiological, or even atomic weapons [1,2,3] (http://www.dni.gov/index.php/nctc-home, accessed on 15 December 2021; https://www.europol.europa.eu/about-europol/european-counter-terrorism-centre-ectc, accessed on 15 December 2021).

Radiation exposure is a danger from both potential “dirty bomb” terrorist events and industrial mishaps including problems with atomic reactors or misplaced radioactive sources. Calamities including exposure to radiological materials require technical planning and readiness to guarantee the health of first responders, the evacuation and clinical therapy of possibly contaminated casualties, and the management of the process of triage. Significant advances have been made throughout the most recent decade in public health and clinical planning intended to improve the response to an atomic explosion or a radiological episode [4,5,6].

A mass victim event would surpass the reaction capacity of the local responders and, subsequently, its methodology would require the mediation of exceptionally well prepared personnel and extensive public activity, based on a fast intervention plan arranged ahead of time. The best model (even though the most improbable) would be the explosion of an improvised nuclear device (IND), which would produce a fireball and a bright glimmer of irradiation followed by an impact wave and thermal pulse. That scenario would make it very hard to get supplies and personnel into the harmed areas, as well as the clearing of the injured to clinics. Mass screening of the affected people would be important to isolate those exposed from non-exposed and to take decisions based on the estimated dose received [7].

Exposure would result from irradiation close to the site of the explosion, which emits radiation at a high dose rate for a brief timeframe; and from deposited radioactivity (also known as aftermath), which has a lower dose rate. The absolute ingested dose would be reliant on the location of the people and the term of their exposure.

The number of individuals exposed, and the dosages received would likewise rely upon a number of factors, e.g., geological characteristics of the area (metropolitan or countryside, protection against radiation provided by buildings), environmental conditions, and the protection set up during the first hours.

Independent of the type of atomic or radiological crisis, explicit (pre-events) plans and reactions should incorporate innovative work in comprehending the pathophysiology of radiation injury, improvement of clinical countermeasures (MCM) (i.e., radioprotectors, radiomitigators, and radionuclide scavengers), and investigating a range of analytic tests to help the clinical decision-makers [8]. Ideally, planning should include energy, health, human management, security, work, transportation, ecology, aeronautics, and atomic guidelines.

## 2. Nuclear and Radiological Accidents

The scenario of the Chernobyl and Fukushima-Daiichi accidents comprised release of large amounts of radionuclides. In water reactors, vaporous and unpredictable splitting of items, specifically isotopes of iodine and cesium, would be determinant for the radiological issues off-site [9], as occurred in Fukushima (https://www.iaea.org, accessed on 15 December 2021). Less unpredictable splitting items or actinides would be critical in case of extreme reactivity accidents (like Chernobyl) in which fuel “hot particles” were delivered [10]. In the primary time frame during the crisis period of an atomic mishap, large amounts of iodine isotopes can reach individuals, with the thyroid being a basic target organ. Triage is critical to distinguish between individuals who need care because of their degree of exposure and those who need health observation. The characterization of the radiological circumstances of individuals and the environment is key to setting up protecting activities (https://www.icrp.org, accessed on 15 December 2021). In the more extended term, contamination of the environment with cesium and other seemingly perpetual radionuclides will influence life in the affected areas, where the external and interior exposure of people ought to be checked to implement effective countermeasures.

The scenario after a huge radiological accident, similar to that which occurred in Goiania (1987) with an enormous ^137^Cs source left in a closed oncologic facility, can likewise be difficult to oversee. In the Goiania accident, four deaths were recorded, 250 people suffered contamination, 62 of them were administered a radionuclide scavenger (Prussian blue), whereas more than 112,000 individuals were radiologically observed, and 3000 m^3^ of radioactive wastes was generated (https://www.iaea.org, accessed on 15 December 2021). Another significant radiological event was that of the ^210^Po poisoning of Aleksandr Litvinenko in 2006 [11], which required follow up of the polonium pollution and screening of more than 750 individuals for their likely internal contamination, thus requiring a huge coordinated effort [12].

## 3. Main Radiations Associated to Nuclear and Radiological Emergencies

Injury from an atomic explosion will fluctuate contingent upon the exposure to various sorts of energy: heat, representing around 35% of total energy; blast, representing roughly half of total energy; and radiation, representing the leftover 15% of energy [13]. Here, the brief acute exposure would be promptly caused by emitted gamma rays combined with a subordinate dose of fast-moving neutrons. Neutrons can represent comprise 25 to 50% of the absolute radiation dose at a distance of approx. 1 km. This is important because, due to its high radiation biological effectiveness (RBE) and radiation weighting factors (WR) (www.icrp.org, accessed on 15 December 2021), the neutron dose can increase multiple times the harm of an equal photon absorbed dose.

The radiation dose received from an atomic blast will be prompt (that delivered with the impact wave), plus an additional relevant component due to fallout of fission and activation products that can be extended (from the aftermath) for a long time as polluted materials tumble to the earth [14]. The mean deadly dose of radiation that would kill half of the people in 60 days (LD50/60), after a total-body irradiation (TBI), is of approx. 3.25–4 Gy in individuals without supportive care; and 6–7 Gy when anti-infection agents and additional support are given [15,16].

In an IND-related event, gamma and neutron radiations will be released, and then gamma and beta radiations from items delivered by the blast [17,18,19].

In a radiological dispersal device (RDD or dirty bombs)-related event, the radiation exposure would be limited, as most likely just one sort of isotope would be utilized. In most RDD scenarios, even with the utilization of solid gamma-discharging radionuclides, huge radiation wounds should not to be normal. The dispersal impacts of the weapon would dissipate the radioactive source [20,21].

An individual exposed to radiation is not radioactive, while an individual contaminated with radionuclides (internal or remotely) may emit radioactivity that is perceptible with hand-held Geiger counters or whole-body scanners. Contamination results when a radioisotope (as gas, fluid, or solid) is delivered to the environment, and afterward ingested, inhaled, or deposited on the body’s surface [22]. A prominent exemption is a neutron radiation exposure, where the cycle of neutron actuation can create biological radioactive material [23].

## 4. Triage and Categorization

The kind of triage varies with the type of radiological or atomic event. For instance, in the case of an atomic explosion, an enormous number of individuals should be assessed, including those affected by a high dose and those having negligible or no actual injury. The dose will be a critical boundary for a clinical triage. As of now, the most productive and available triage technique is the utilization of consecutive complete blood counts to evaluate lymphocyte exhaustion that is associated with assessed whole-body dose radiation exposure. If fast blood testing would not be conceivable, dose assessment can be at first evaluated dependent on basic boundaries, i.e., correlations between the extent of the body exposed to the radiation and the % of the radiation levels estimated in the environment; victim’s shielding activities after the explosion; and signs and side effects from exposure to radiation or early radioactive particles’ aftermath [24]. The radiation dose classes allude to dosages affecting the whole body or a large portion of the body (partial exposure). Notwithstanding the straightforward boundaries referenced above, the dose can be additionally be assessed dependent on: (a) the period until onset of early signs, (b) the seriousness of the signs (i.e., the acute radiation syndrome, ARS), and (c) the biodosimetry methods [25,26,27]. Even though vomiting is a serious basic symptom after whole or huge partial body radiation exposure, it cannot be utilized to anticipate the radiation dose received. Vomiting can likewise be brought about by head injury, uneasiness, or other pathology [28].

It is key to point out that viable clinical triage can save numerous lives. In this, a fast reaction, sufficient coordination, and the accessibility of innovative biodosimetry is required. Clinical triage following an atomic explosion ought to be a stepwise cycle, where the principal point is abbreviated as “SALT”- Sort, Assess, Lifesaving Interventions, Treatment/Transport [29]. In the military, operational organizers use ‘parts’ to characterize the four levels where military clinical help is coordinated on a reformist premise to lead triage evaluation, quick treatment, evacuation, resupply, and capacities basic to the upkeep of health [30]. Stepwise triage should incorporate the point of care (POC) evaluation (blood counts, see above), followed by secondary evaluation, perhaps with high throughput screening to additionally characterize a person’s dose (so that individuals considered in danger of showing ARS throughout the following weeks are identified). Also, assays which could be utilized for assessing long-haul malignant growth hazards (for example quality screening) ought to likewise be incorporated [31]. It is also imperative to consider that amid a radiological or atomic crisis, where the coordination of numerous individuals and management is fundamental, an unmistakable and agile command chain is vital.

## 5. Biological Effects in Nuclear and Radiological Accidents

### 5.1. Oxidative Stress and Inflammation at the Core of Ionizing Radiation-Induced Damage

Ionizing radiation (IR) can break covalent bonds and cause oxidative harm to DNA, lipids, proteins, and numerous metabolites [32]. In experimental processes it is shown that the DNA molecule is more radiosensitive when it is irradiated in solution than in a dry environment [33]. The effects of IR on the DNA molecule are single and double chain fractures, structural alterations and elimination of the bases generating apurinic and apyrimidinic sites (AP sites), sugar damage, cross-links between DNA-DNA or between DNA-protein, and breaking of hydrogen bonds [34,35]. Moreover, overproduced reactive oxygen species (ROS) can react with cell membrane fatty acids and proteins impairing their function [36]. The primary event for the formation of a free radical in the radiolysis of water is the release of an electron in the interaction of low linear energy transfer (LET) ionizing radiation with the water molecule [37]. While the physicochemical events are a quick result of radiation exposure, the damage propagates the reaction by producing repeating waves of ROS, reactive nitrogen species (RNS), cytokines, chemokines, and other factors with related incendiary penetration [38].

During the radiolysis of water, ROS like superoxide anion (O_2_^•−^), hydroxyl radical (^•^OH), hydrated electron and hydrogen peroxide (H_2_O_2_) are produced [39]. The release of nitric oxide (NO^•^) and its metabolites such as peroxynitrite (ONOO^−^) and nitrogen dioxide (NO_2_^•^) are also involved in IR genomic damage [5]. Overproduction of ROS and RNS is a harmful process that can cause damage to cellular biomolecules (DNA, proteins, and lipids), and affect the cell membrane, cellular signaling and genome integrity. These effects can influence numerous cellular processes linked to cell death, carcinogenesis, and cancer progression [40,41,42]. Indeed, oxidative stress, and the associated redox status shifts, can cause cell transition from quiescent to proliferative status, growth arrest or cell death activation according to the duration and extent of the redox imbalance [43]. In turn, cells injured by IR are responsible for inducing radiation bystander effects (RIBEs) in non-irradiated cell recipients, manifested by changes including (but not limited to) gene expression, protein synthesis, chromosomal aberrations, micronuclei formation, secretion of exosomes and miRNAs, and cell death/proliferation or transformation [44,45,46]. ROS are considered initiators, and NO, the transforming growth factor beta (TGF-β) and other inflammatory cytokines effectors are involved in RIBE [47,48]. Moreover, the inflammatory response generates recurring waves of ROS, cytokines, chemokines and growth factors with associated inflammatory infiltrates [49,50]. This represents a vicious circle where both oxidative stress and inflammation induce each other. These concepts are supported because non-steroidal anti-inflammatory drugs and antioxidants decrease some of that latent damage, as well as the inflammation-associated mutations. This is a crucial point that determines that MCM to reduce the damage induced by IR is based on free radical scavengers, antioxidants, and anti-inflammatory agents [51,52,53,54].

### 5.2. Acute and Chronic Radiation Syndromes

Biological impacts will fluctuate contingent upon the type and dose of radiation, and the time and recurrence (single or serial) of exposure [55]. The impacts of radiation on the body may show up rapidly (acute radiation syndrome, ARS) or require several years after exposure (deferred impacts, for example, fibrosis, sterility, genetic impacts, or malignancies). By and large, exposure to higher doses of radiation produces symptoms more quickly [54]. In the case of an atomic impact, radiation-derived wounds will be of different types, for example, injuries or thermal burns [56]. Heat and light cause thermal injury, including flash burns, fire burns, flash blindness (because of transitory loss of photopigments from retinal receptors), and retinal burns. The impact wave can cause breaks, slashes and cracks of the viscera, and aspiratory drain and edema [56].

Non-deadly harm (mis/unrepaired) may prompt genomic unsteadiness, for example, chromosomal variations, DNA mutations, and cell senescence. According to radiation assurance measures, radiation-prompted impacts are classified as (a) deterministic (tissue responses which require a threshold dose to exceed) which result from cell execution or the deficiency of cell capacity; and (b) stochastic or irregular (not relying upon such a limit, although its likelihood increases as the radiation dose expands) which are brought about by hereditary deviations and mutations setting off long term inherited impacts and malignancies [57].

The Life Span Study (LSS, https://www.rerf.or.jp, accessed on 7 January 2022) is an exploratory program examining deep-rooted health impacts dependent on epidemiologic (accomplice and case-control) considerations. Its most significant target is to explore the longer term impacts of bomb-derived radiation on reasons for death and the occurrence of malignancy. The examination has indicated that the danger of solid malignancy and leukemia among atomic specialists is steady with the dosage assessed, even if they get the radiation at low dose rates over numerous years [58]. The global INWORKS study has shown that in any event, when the combined dose of atomic industry laborers was under 100 mSv and the dose rate was under 10 mGy every year, the danger of solid malignancy is steady based on the dosage assessment [59]. A recent review [60] identified a large body of epidemiological data (published between 2006–2017) that assesses the evidence of an increase in solid cancer risks and/or leukemia, following low-dose IR exposure (<100 mGy).

ARS involves different phases of biological injury that may follow exposure (of the whole body or its majority) to a high dose of radiation (ordinarily in a brief timeframe). Its seriousness relies upon the radiation dose and normally includes syndromes whose term is directly correlated with the total dose received (and, ultimately, with the pace of exposure) [61,62,63,64]. Initially, a prodromal phase may show up with side effects, for example, sickness, spewing, and torpidity. This is continued (in hours to weeks) by various conceivable subsyndromes (related to various dose limits) for example the hematological (at doses of 1–2 Gy), gastrointestinal (GI) (dosages of 4–6 Gy), cutaneous (approx. 6 Gy), cerebrovascular (approx. 10 Gy) [65,66]. Lung wounds (approx. 8 Gy) may likewise show up half a month after exposure. An idle period of hematological ARS may infer a time of 1-3 weeks after getting a total dose of 2–4 Gy. Higher dosages may abbreviate or eliminate the inert phase [66].

Chronic radiation syndrome (CRS) results from long-term repeated exposure (external and/or radionuclide intake) to rather low doses [from 0.7–1.5 Gy (at rates > 0.1 Gy/year) to 2–3 Gy] and has a long-term intermittent course. It is worthwhile to point out that cancer induction can also be found at lower doses (<0.7 Gy). In the beginning, it was considered that CRS manifestations could also include the chronic ARS damages, but as tissue reaction mechanisms of ARS and CRS differ, such association was recognized as incorrect [67]. The CRS term does not refer to the duration of disease (ARS manifestations can also remain for a long time, and develop chronic pathologies), but characterizes the result of protracted (chronic) radiation exposure [67,68,69].

Initial CRS symptoms are nonspecific, and can be reversible, if there is a decrease or a break in radiation exposure. If exposure continues, the initial symptoms grow progressively worse, and others may appear. The earliest manifestations of CRS are a dose-dependent inhibition of hematopoiesis and neurologic dysfunctions. Moderate but persistent leukopenia induced by neutropenia is one of the typical changes in peripheral blood, although in certain patients lymphopenia was also noted [68,70]. A severe degree of CRS is characterized by the development of bone marrow hypoplasia, persistent and marked granulocytopenia, profound thrombocytopenia, and moderate anemia. In these cases, hematopoiesis recovery is quite difficult or even impossible, even though the radiation exposure is discontinued [69]. Three sequential neurologic syndromes have been identified: vegetative dysfunction with impairment of neuro-visceral regulation, asthenic syndrome, and encephalomyelitis-type lesions of the central nervous system. Neurosensitive dysfunctions (olfactory and vestibular excitability decline, taste fatigue, etc.) sometimes precede the neuro-vegetative syndrome which is considered the earliest manifestation of the CRS [70]. Signs of vegetative dysfunction include: decrease in capillary tone (especially in skin vessels), an intense histamine-induced skin reaction, instability of the pulse with a tendency to hypotension, changes in the secretory and motor activity of the GI tract, etc. [70]. Some women develop changes in the sex hormone ratios (total estrogen levels were found at the lowest limit), in most cases accompanied by menstrual cycle disorders [69]; in animal models, a reduced number of follicles have been evidenced [71]. The rate of spontaneous abortions was five times higher than that without exposure [72]. The asthenic syndrome has a gradual progression, i.e., fatigue, headaches, dizziness, general weakness, hypersomnia, decreased working capacity and considerable memory deterioration [69,73]. At this stage patients can suffer cataracts, skin disorders such as a decrease in elasticity, dermatitis, xeroderma or hair loss [74]. Vascular dysfunction and thrombocytopenia play a key role in predisposition to hemorrhagic events like cutaneous petechial, mucosal, and visceral hemorrhages. Functional activity of organs and tissues, as well as structure, can undergo considerable changes (fibrosis, hypoplasia, malignant transformation, etc.). Radiation-related risk of cardiovascular disease is increased and can be associated with lung and heart fibrosis and atherosclerotic disorders [75]. Quite often, the CRS of medium severity is complicated by infections of respiratory and digestive systems [69,73]. When a demyelinating encephalomyelitis is developed, the patients’ health status deteriorates dramatically, accompanied by general weakness and adynamy [69]. Although the brain has been classically regarded as a radioresistant organ, vascular lesions (edema, thrombosis, hemorrhage) and Blood-Brain Barrier (BBB) disruption are considered to be a precipitating factor for white matter necrosis [76]. Causes of death in the late period of CRS are sepsis and hemorrhages resulting from inhibition of hematopoiesis and immunity, malignant solid tumors and especially leukemia and chronic myeloleukemia [73]. There is evidence that relative risks are generally higher after radiation exposures in utero or during childhood [77].

Hereditary harm brought about by radiation is behind the expansion in the recurrence of malignant growths and can show both in the early phases and throughout the long term. As a reasonable model, notwithstanding acute ailments, numerous survivors of Chernobyl, Nagasaki, and Hiroshima additionally endured leukemia, and thyroid, stomach, and skin malignant growths (https://www.unscear.org, accessed on 7 January 2022) [74]. Studies on the nuclear bomb survivors in Japan revealed that the danger of mortality of solid malignant growth became apparent approximately ten years after detonation and expanded by half when the dose to which the colon was exposed arrived at 1 Gy; the danger of mortality from leukemia was quadrupled when the dose to which the red bone marrow was exposed reached 1 Gy [78,79,80].

## 6. Medical Countermeasures

It is critical to develop effective radioprotectors as a preventive measure for their application in planned radiation usage, such as radiation therapy, as well as unplanned exposure, such as natural background radiation, space travel, nuclear disasters, and nuclear warfare. The IR research program of the US National Cancer Institute proposed the following pharmacological classification of agents with IR protection properties according to the timing of administration: (a) protection, (b) mitigation, and (c) therapeutic agents [81]. In general, radioprotectors are used before IR exposure to protect cells and tissues from being damaged; radiomitigators are administered during or shortly after IR exposure, and attenuate damage and/or contribute to tissue recovery. Lastly, therapeutic agents are administered after symptoms have presented, acting as palliation or support [82]. As we will explain below, due to the capacity to scavenge free radicals, some antioxidants can be considered as radioprotectors, and many of them act also as radiomitigators for their capacity to enhance cellular antioxidant and repair mechanisms, during and after IR exposure. Finally, only a few can also be considered as therapeutic agents by reducing or palliating the clinical symptoms induced by exposure to IR.

The improvement of viable MCM to shield individuals from the unsafe impacts of normal radiation constitutes a neglected need [54]. Considering explicitly the radiological or atomic crises where earnest assistance is required, it is critical to plan separately for first responders, and for those directly presented to radiation during the mishap. First responders’ vulnerability may be reduced by radioprotectors and radiomitigators, while those exposed to radiation may require radiomitigators, and of course, therapeutic support.

### 6.1. Radioprotectors

An ideal radioprotective agent should fulfil several criteria, i.e., provide significant protection, be stable, offer the chance of a simple formulation, have an easy route of administration, and have no significant toxicity (mainly in particularly sensitive tissues, in which acute or late toxicity would be dose restricting). No single molecule so far has every one of these properties, and at this moment, radiation MCMs for ARS and other exposure-related injuries are assigned FDA orphan drug status [83].

Many different molecules have been assayed as potential radioprotectors. Some show promising properties but, considering pharmacokinetic properties and ease of in vivo administration, we might suggest the following for a potential radioprotective formula:

#### 6.1.1. Thiol-Containing Compounds

Since the detonation of the Hiroshima and Nagasaki bombs, the Walter Reed Army Research Institute (USA) enhanced its research program on radioprotective countermeasures and screened more than 4000 compounds [84]. Cysteine was the first one to confer radiation protection in mice subjected to total body radiation (TBI) [85], and since then many synthetic aminothiols have been developed and proved. Undoubtedly, the most effective was WR-2721 or amifostine, a sulfhydryl prodrug activated by alkaline phosphatase to the active WR-1065. Salivary glands and the epithelial cells of intestine are highly enriched in this activating enzyme, and thus oral administration of WR-2721 just before radiation results in localized high production of the bioactive derivate, preventing radio-induced mucositis and GI damage without significant systemic side effects [86,87,88,89]. The underlying mechanisms of action are free radical scavenging and hydrogen atom donation, along with DNA protection and repair; all coupled to an initial induction of cellular hypoxia [90,91,92]. WR-1065 has anti-mutagenic and anti-carcinogenic properties evidenced using in vitro testing systems [91], induces G1 cycle-arrest and p53 dependent-cytoprotection [52], upregulates the expression of mitochondrial Mn-SOD2 and proteins responsible for DNA repair, and inhibits apoptosis through Bcl-2 and hypoxia-inducible factor-1α (HIF-1α) [87]. Amifostine was the first Food and Drug Administration (FDA)-approved clinical radiation protector intended to reduce the impact of radiation on normal tissue, and more specifically, to decrease xerostomia in patients undergoing radiotherapy for head and neck cancers [92]. WR-1065 accumulates more rapidly in normal tissues than in malignant cells, due to the relative lower activity of alkaline phosphatase in cancer cells and acidic pH in the environment of many tumors. Amifostine is clinically used to prevent xerostomia, mucositis, dysphagia, dermatitis, and pneumonitis during radiotherapy of head and neck cancers, and a meta-analysis carried out in 2014 pointed out its beneficial effects [88]. However, a more recent randomized double-blind trial [89] does not support any benefit. Despite the heterogeneity, results appear to show some benefit to its use as radioprotector [87].

The glutathione redox status (GSH/GSSG) decreases after irradiation, mainly due to an increase in glutathione disulfide (GSSG) levels. Two reasons may explain the radiation-induced increase in blood GSSG: (a) GSH reacts with radiation-induced free radicals forming thiol radicals that react to produce GSSG; and (b) GSSG is released from different organs (e.g., the liver) into the blood. In fact, GSH is essential to prevent radiation damage and the glutathione redox ratio in the blood can be used as an index of radiation-induced oxidative stress [93]. The DNA single-strand breaks repair system is absent in GSH-deficient cells, and GSH is also essential to activate proliferation and repair of damaged tissues and to prevent cell death [94]. In fact, the main mechanism of action of most radioprotectors is to maintain intracellular levels of GSH. An illustrative example is *N*-acetylcysteine (NAC), a potent antioxidant and GSH precursor. NAC treatments (300 mg/kg, sc), starting either 4 h prior to or 2 h after radiation exposure, and with six subsequent daily injections over 7 days, reduced early deaths in abdominally irradiated (X-rays, 20 Gy) C57BL/6 mice [95]. More recently, radioprotective effects of NAC have been demonstrated in multiple studies [96,97], but the use of GSH or NAC with oncoradiotherapy cannot be supported because it may also favor cancer cell metastasis and radioresistance. Erdosteine (a homocysteine derivative) is a potent free radical scavenger, increases GPx and catalase (CAT) activities and GSH intracellular levels. Erdosteine treatment before γ-radiation ameliorated nephrotoxicity, and decreased IL-1, IL-6, and tumor necrosis factor alpha (TNF-α) blood levels, thus suggesting substantial protection against radiation-induced inflammatory damage [98].

Aminothiols and their phosphothioate derivatives, administered shortly before irradiation, exert radioprotection by one or a combination of effects: scavenging of radiation-induced free radicals; induction of hypoxia; formation of mixed disulfides; quenching of metals; repair of DNA and genome stabilization. However, radioprotectors of this type, including amifostine, have important side effects and a short pre-exposure time window of radioprotectiveness, which limit their use as radiation countermeasures [92,99]. Any strategy aimed at reducing toxicity, without reducing their radioprotective efficacy, would be a great advance. Rather novel approaches include: (a) slow-release delivery of drugs, (b) combined treatments with other radioprotectors/radiomitigators such as cytokines (G-CSF), selenium, metformin, antioxidants, etc.; (c) re-engineering better tolerated analogs like HL-003 or combining with antiemetic drugs; and (d) molecular conjugates and nanoparticle formulation designed to extend amifostine or WR1065 circulating half-life or to avoid intravenous administration. As reviewed by Singh y Seed [92], these approaches have proven to be useful but without a complete elimination of the toxicity or just increasing the radioprotection to a limited extent.

#### 6.1.2. Natural Phytochemicals

Over the last decades many phytochemicals, and especially polyphenols, have been broadly considered as radioprotectors and/or radiomitigators. The antioxidant activity of polyphenols depends, in part, on their ability to delocalize electron distribution, resulting in a more stable phenoxy group. Thereby, differences in ROS scavenging potential can be attributed to the different functional groups attached to the main nucleus [100]. Intercalation in DNA double helices induces stabilization and condensation of DNA structures making them less susceptible to free radicals’ attack [100], reducing genotoxic damage induced by IR [101]. Xanthine oxidase and lipoxygenase are inhibited by many polyphenols, thus reducing the generation of free radicals. Finally, many polyphenols decrease the activation of NF-κB and MAPK, thus reducing the release of inflammatory cytokines which play a role in the radiation-induced inflammatory response [102,103,104].

Genistein nanoparticles increase the expression of metallothionein genes and suppress the post-irradiation increase of cytokine production (IL-1-beta, IL-6) and cyclo-oxygenase-2 (COX-2) activity, thus preserving bone marrow progenitors and increasing survival on day 7 post-irradiation (9.25 Gy ^60^Co) [105]. The radioprotective effects of genistein are due to its ability to inhibit NF-κB, MMPs, and Bax/Bcl-2 signaling pathways and attenuate the inflammatory response induced by IR. In rodents, genistein has been shown to mitigate the effect of radiation on the lungs [106] and the intestinal tract [107]; used in combination with radiotherapy in prostate cancer patients, it can reduce intestinal, urinary, and sexual adverse effects.

The positive effects of curcumin as a radioprotector involve its free radical scavenging activity, antioxidant properties targeting the Nrf2 pathway [108], and its anti-inflammatory effects mediated by modulation of COX-2, IL-1, IL-6 [109], tumor necrosis factor alpha (TNF-α), TGF-β expression, release and/or activity [110,111]. Curcumin ameliorated radiation-induced pneumonitis and pulmonary fibrosis [112,113] and cognitive deficits (including learning and memory defects), exerted cardioprotective, neuroprotective, hepatoprotective, and renoprotective activities [108,110], and decreased pain severity [114]. Additionally, curcumin has antitumor effects [115] that can synergize with radiotherapy [116,117,118]; it should thus be considered a good option to increase the efficacy of radiotherapy on cancer cells, as well as to prevent the radiotherapy-induced adverse effects in normal tissues [112,114]. A few human studies have confirmed its efficacy for the management of radiotherapy induced dermatitis [119] and mucositis [120,121]. To modify the pharmacokinetic profile of curcumin and increase its bioefficacy, new formulations have been introduced [122].

Epigallocatechin-3-gallate (EGCG) and other flavonoids from green tea inhibited radiation-induced damage [123]. EGCG scavenges free radicals, increases the levels of several antioxidant enzymes, i.e., glutamate-cysteine ligase, SOD, and heme oxygenase-1 [124,125] and induces Nrf2 activation which, in turn, represses radiation-induced apoptosis and attenuates TBI-induced intestinal injury [126]. The inhibition of the proteasome, a regulator of inflammation, has been reported as well and, consequently, extracts of green tea decreased the release of pro-inflammatory cytokines, i.e., TNF-α, PGE2, IL-1β, IL-6 and IL-8 in vivo [127]. Epicatechin blocked ROS production and radiation-induced apoptosis via down-regulation of JNK and p-38, which ameliorated oral mucositis and survival rates [128], inhibited radiation-induced auditory cell death of rats [129], and enhanced the recovery of hematopoietic cells in mice [130].

Resveratrol (RES) has demonstrated potential anti-cancer, antioxidant, neuroprotective, anti-inflammatory and cardioprotective effects. It is noteworthy that RES serves as a scavenger of O_2_^•−^, ^•^OH and metal-induced radicals, and increases the activity of many antioxidant enzymes [131]. RES significantly reduced radiation-induced chromosome aberration [132], DNA damage [133] and apoptosis, supported cell regeneration, and induced repression of the NLRP-3 inflammasome subset [134]. In mice, administration of RES attenuates radiation-induced intestinal damage via activation of sirtuin-1 [135], supporting lymphocyte [136] and intestinal functions recovery [137]. Under oxidative stress, RES promotes tyrosyl-tRNA synthetase acetylation, regulates relevant signaling proteins, and reduces apoptosis and DNA damage [138]. Clinical studies on RES as a normal tissue protector and potential tumor sensitizer are limited [139], mainly because RES possesses unfavorable bioavailability and pharmacokinetic properties. Synergistic effects with other polyphenols such as curcumin have also been evidenced and new formulations (hybrid molecules or nanoparticles) are being tested to increase its bioavailability and efficacy [140]. The use of derivatives, such as pterostilbene, with similar properties and a longer biological half-life, can significantly contribute to improve the radioprotective effects in vivo, as we have evidenced in our laboratory [141].

Oral silibinin treatment (100 mg/kg/day) reduced late-phase pulmonary inflammation and fibrosis in C57BL/6 mice after 13 Gy thoracic irradiation, via downregulation of NF-κB [142]. We have reported synergic radioprotective effects of silibinin with pterostilbene, resulting in 100% of the mice surviving, 30 days after TBI g-irradiation of 7.6 Gy (LD50/30) [141]. Silibinin can chelate thorium radionuclides (^232^Th) preventing hemolysis and enhancing liver cells decorporation, which is important because those cells are the major targets of internalized ^232^Th [143].

Quercetin minimizes radio-induced oxidative damages and genotoxicity, preventing hematopoietic genomic instability and dysfunction [144] and skin fibrosis [145]. Quercetin pre-treatment attenuated ROS generation, downregulated NF-κB and reduced expression of proinflammatory cytokines (PGE2, IL-1β, IL6, IL-8 and TGF-β) [146]; it also reduced DNA double-strand breaks and cellular senescence in C57BL/6 mice exposed to a single-dose (25 Gy) or fractionated IR doses [147]. The anti-inflammatory effects of quercetin are also favored by its ability to reduce recruitment of neutrophils, myeloperoxidase and COX-2 activity, MAP kinases signaling and NLRP3 inflammasome activation in macrophages [148].

Recently, Faramarzi et al. [103] reviewed the radioprotective potential of natural polyphenols and, based on their dose-dependent antioxidant/pro-oxidant efficacy, concluded that they could represent a valuable alternative to synthetic compounds. Polyphenols provide protection to normal cells, with little or no protection to cancer cells, and in some cases, have the additional advantage of increasing cancer radiosensitivity. The potential use of polyphenols as radioprotectors is based on their low toxicity, the suitability of oral administration, and the possibility of combining several of them. Nevertheless, their low bioavailability due to poor absorption, rapid metabolism, and/or rapid systemic elimination, can compromise their efficacy. Thus, new pharmaceutical formulations (nanoparticles, vesicles, cocrystals…) are being implemented and tested to facilitate oral administration and/or increase their effectiveness (see, e.g., https://www.circecrystal.com, accessed on 21 January 2022) [149,150,151].

The most promising non-polyphenolic phytochemicals with radioprotective effects are sesamol, gallic acid and caffeic acid derivates. The strong antioxidant activity of sesamol has been reported in comparison to standard antioxidants like vitamin C, curcumin, etc. Sesamol pre-treatment at 50 mg/kg (oral) was found to be the most effective dose in reducing mortality in irradiated Swiss albino mice exposed to 9.5 or 15 Gy γ-TBI [152]. The radiation-induced increase of apoptotic biomarkers and decrease in endogenous antioxidants (GSH, GST, CAT) was reduced by sesamol treatment, preserving crypt cells, villus height, and intestinal [152] and hematopoietic functions [153]. A recent study evidenced that daily oral consumption of sesamol is more effective than administration of a single dose before irradiation [154]. Similar results were observed using 100 mg/kg of gallic acid 1h prior to 10 Gy radiation exposure [155]. The cytoprotective effects of gallic acid are also due to its ability to enhance DNA repair, chelate metal ions, through the attenuation of MAPK and NF-κB/AP-1 signaling pathways, and reduce the release of inflammatory cytokines and adhesion molecules involved in leukocyte infiltration [156]. Caffeic acid (CA) and caffeic acid phenethyl ester (CAPE) act as free radical scavengers, compete with oxygen for IR-induced electrons, have antioxidant effects [151,157,158], decrease lipid peroxidation and increase antioxidant defenses in the heart and lung tissue of irradiated mice [159]. Treatment with CAPE prior to irradiation of rats effectively ameliorated intestinal [160], and hepatic [161] injuries. CA and CAPE inhibit activation of NF-kB, VEGF secretion and COX-2 activity, being considered potent anti-inflammatory agents [159,162]. In addition, CA stimulates cell cycle arrest and increases cell death in tongue, neck, and mouth cancer cells [158] and both molecules have anticarcinogenic properties attributed to their capacity to reduce tumoral angiogenesis, cancer growth and metastasis progression [158,162,163]. CAPE is a lipophilic agent, but incorporation into nanoparticles facilitates its administration. Moreover, nanoparticles can be modified to respond to different stimuli, such as pH, temperature, magnetic fields, oxidative stress, irradiation etc., thus facilitating the sustained release of drugs in selected tissues. That is the reason why even though CAPE-nanoparticles showed a similar protective activity compared to CAPE under in vitro conditions, mice treated with nanoparticles had a longer survival after being exposed to IR [151].

Dietary sources of phytochemicals mentioned in this article and their radioprotective properties are detailed and reviewed in [102,103,104,164].

#### 6.1.3. Vitamins

With the understanding that free radicals perpetuate a significant amount of the damage caused by IR, vitamins with antioxidant potential (A, C, and E and its derivates) have been assayed as radioprotectors. Vitamin A and carotenes have antioxidant activity and capacity to enhance DNA repair, and in vivo reduced mortality and morbidity in mice exposed to partial or TBI [165]. Carotenoids such as crocin and crocetin (isolated from saffron) have antioxidant, anti-inflammatory and antiapoptotic effects [166]. In mice bearing pancreatic tumors, crocin significantly reduced tumor burden and radiation-induced hepatic damage [167], while crocetin reduced in vitro radiation injury in intestinal epithelial cells [168] and testis injury in pubertal mice exposed to 2 Gy X-rays [169]. Lycopene is the carotene isomer with the highest antioxidant potential and capacity to reduce proinflammatory cytokines expression such as IL-8 and IL-6 or NF-κB. Pre-clinical studies evidenced its radioprotective efficacy, particularly, if it is administered previously to or as soon as possible after radiation exposure [170,171] which is very interesting because lycopene has also anti-cancer activity, as recently reviewed in [172].

Administration of vitamin C (ascorbic acid, AA) before g-irradiation prevents chromosomal damage in bone marrow cells, mainly due to its antioxidant activity [173], reduces the GIS severity [174] and the adverse effects of TBI in the liver and kidney [175]. Moreover, intraperitoneal administration of 3 g AA/kg, up to 24 h after TBI (7.5 Gy), significantly increased survival in mice, reduced radiation-induced apoptosis in bone marrow cells, and restored hematopoietic function [176]. Nevertheless, administration of less than 3 g AA/kg was ineffective, and doses of 4 or more g/kg were harmful to mice. Moreover, treatments beyond 36 h were ineffective [176]. These facts highlight the limited efficacy margins of the treatment and compromise its use as a radioprotective measure.

Vitamin E is an essential fat-soluble nutrient with antioxidant, anti-inflammatory and neuroprotective properties. Eight vitamers are included in the vitamin E family, four saturated (α, β, γ, and δ) called tocopherols, and four unsaturated analogs (α, β, γ, and δ) referred as tocotrienols [177]. All of them are collectively known as tocols, and α-tocopherol is the most abundant in human tissues. Tocols are free radical scavengers, potent antioxidants and anti-inflammatory agents with capacity to attenuate fibrosis in tissues exposed to IR [177,178,179]. α-tocopherol succinate inhibited radiation-induced apoptosis and DNA damage, increased antioxidant enzymes activity, protected active mitotic tissues, and inhibited the expression of oncogenes in irradiated mice [180]. Moreover, when *α*-tocopherol was administered 24 h before ^60^Co *γ*-radiation, there was a significatively increase in the survival rate of mice, attributed to the capacity to restore crypt cellularity and inhibit bacterial translocation from the gut to the bloodstream [181]. Further studies revealed that α-tocopherol succinate significantly reduced thrombocytopenia, neutropenia, and monocytopenia, an effect mediated through induction of high levels of granulocyte colony-stimulating factor (G-CSF) [182]. Moreover, pre-clinical studies provided evidence that tocotrienols radioprotection is exerted, in part, via induction of G-CSF [183,184], suppressing expression of TNF-α, IL-6, IL-8, inducible nitric oxide synthase (iNOS), and NF-κB signaling [179]. IR downregulates the expression of thrombomodulin (TM) and increases endothelial surface expression of adhesion molecules which allow the attachment of immune cells and, thereby, contribute to inflammation and activation of the coagulation cascade. In this regard, the efficacy of tocotrienols is attributed to their higher antioxidant potential, their ability to inhibit HMG-CoA reductase activity [185], and increase TM expression in endothelial cells [186], which result in anti-permeability, anti-inflammatory and anti-thrombotic response [179]. Promising radioprotective results of γ-tocotrienol (GT3) have been demonstrated in mice [187] and primate models, by preserving the hematopoietic stem and progenitor cells, and recovery from γ-irradiation (5.8 or 6.5 Gy)-induced neutropenia and thrombocytopenia [188,189]. Recent preclinical studies evidenced that GT3 may be a potential countermeasure against late degenerative tissue effects of high-LET radiation in the heart [190] and lung radiation injury [191]. Tocotrienols accumulate in the intestine to a greater level than tocopherols, and this can be involved in its greater ability to attenuate GIS [192]. γ-tocotrienols seem to have a greater efficacy as radioprotectors attributed [189] to their: (a) higher antioxidant potential [191], (b) capacity to downregulate proapoptotic/antiapoptotic ratio [193], (c) ability to accumulate in endothelial cells and intestinal epithelium which facilitates the recovery of mesenchymal immune cells [192], and (d) ability to inhibit HMG-CoA reductase, helping to avoid chronic inflammatory responses associated to radio-induced vascular and intestinal damage [185]. Moreover, recent studies have also evidenced the anti-cancer properties of γ-tocotrienols [179] and, although their low bioavailability is an important limiting factor [177], new formulations may help to overcome this pitfall. In this sense, a novel water-soluble liposomal formulation of γ-tocotrienol selectively targets the spleen and bone marrow with high efficiency, and facilitates rapid recovery of hematopoietic components after lethal TBI radiation in mouse models [194]. High doses of tocols are required to exert radioprotective effects, which increase the risk of toxic accumulative side effects. To ameliorate this risk, several trials have assayed and evidenced additive/synergistic effects with other radioprotectants such us aminofostine [195], simvastin [196], and others. For instance, pentoxifylline (a xanthine derivative approved by the FDA as a phosphodiesterase inhibitor, with antioxidant and anti-inflammatory effects) improved survival and enhanced the radioprotective properties of γ-tocotrienol on the hematopoietic, GI and vascular systems in mice subjected to 12 Gy ^60^Co γ-irradiation [197]. A Phase II clinical trial also demonstrated the radioprotective efficacy of the combination pentoxifylline+vitamin E to attenuate radiation-induced fibrosis [198]. Two randomized controlled trials provided evidence that dietary supplementation of alpha-tocopherol and beta-carotene during radiation therapy could reduce the severe adverse effects of treatment, but also warned that high doses might compromise radiation treatment efficacy [199,200]. Other radioprotective combinations, such as α-tocopherol acetate and AA, had the additional advantage of enhancing apoptosis in irradiated cancer cells [201,202].

Calcitriol upregulates the expression of SirT1, SODs and GPxs and induces the synthesis of metallothioneins in vitro [203,204]. Jain et al. (2013) showed a positive link between vitamin D and GSH concentrations, as well as a reduction in the levels of pro-inflammatory cytokines [205]. Inhabitants of contaminated regions near Chernobyl had lower vitamin D blood levels compared to those living in uncontaminated regions [206]. Therefore, oral supplementation with vitamin D during radiotherapy or in professionals chronically exposed to low IR doses could be doubly useful, preventing radioinduced oxidative stress and osteoporosis [207]. Recent studies evidence that calcitriol selectively radiosensitizes cancer cells by activating the NADPH/ROS pathway [208].

#### 6.1.4. Antioxidant Enzyme Activities and Oligoelements

Many antioxidant/defense enzymes, such as SODs, GPxs, and metalloproteins require trace elements as cofactors (e.g., Cu, Mn, or Se), thus, their dietary supplementation has been widely evaluated as a radioprotective strategy [54,209]. As cofactor for selenoenzymes, i.e., GPxs, thioredoxin reductase-1 and ribonucleotide reductase, Se supplementation enhances GPxs activity, thus reducing intracellular H_2_O_2_ and organic peroxide levels. Both sodium selenite and selenomethionine, i.p. injected before or shortly after (+15 min) radiation exposure (^60^Co, 9 Gy), enhance the survival of irradiated mice, but selenomethionine had lower toxicity [210]. Se treatment enhances Nrf2 transcription and upregulates the adaptive response to IR in bone marrow and hematopoietic precursors [211]. 3,3′-diselenodipropionic acid (DSePA) had maximum absorption in the lung, suppressed NF-kB/IL-17/G-CSF/neutrophil axis and significantly reduced infiltration of neutrophils and levels of IL1-β, ICAM-1, E-selectin, IL-17 and TGF-β in the bronchoalveolar fluid, prevented pneumonitis and increased survival of irradiated mice without affecting radiation sensitivity of tumors [212]. During the reaction with oxidizing free radicals DSePA generates intermediates with GPx like activity that reduce lipid peroxidation, apoptosis and excessive inflammatory response in radiosensitive tissues such as lung, liver, spleen, and GI tract, increasing survival against supra-lethal doses of γ-radiation [213]. Se compounds are less effective than aminofostine as radioprotectors, but have also lower toxicity and can be used in combined treatments [214].

Two consecutive systematic reviews, carried out between 1987 and 2012 [215] and 2013 and 2019 [216] evidenced that cancer patients tend to have low Se blood levels, which is aggravated by radiotherapy and/or its side effects (vomiting, etc.), and associates to a decrease in the activity of different antioxidant enzymes. Based on the results from clinical trials in patients who underwent radiotherapy, it was concluded that Se supplementation prevented or reduced the side effects of radiotherapy without compromising its anticancer efficacy; and consequently, authors highly recommend sodium selenite (200–500 μg/daily) oral supplementation [216]. On the other hand, it is paradoxical that several studies have demonstrated Se can act as prooxidant in a dose dependent fashion and can attenuate DNA repair mechanisms as well as antiapoptotic genes in some cancer cells, being nowadays assayed as a radiosensitizer in oncoradiotherapy. In vivo, the variability in redox potential gradients, the lower pH and the redox imbalance existing in the cancer microenvironment can facilitate the conversion of Se nanoparticles (SeNPs) into a pro-oxidant agent causing mitochondrial dysfunction, cell cycle arrest, and ultimately cancer cell death [217]. Organic Se compounds and especially SeNPs are better candidates as radioprotectors and radiosentitizers for their lower toxicity and higher cancer cell selectivity compared to sodium selenite [217,218].

SODs exist as CuZnSOD (cytosolic and nuclear fraction) and mitochondrial MnSOD, and both scavenge O_2_^•−^ by accelerating its conversion to H_2_O_2_. Attempts to supplement the activity of endogenous SOD include the induction of in vivo gene expression using adenovirus or plasmid liposomes, and administration of nanozymes with SOD-like activity [219]. A porphyrin-mimetic of the human MnSOD (BMX-001), which crosses the BBB, protected the brain’s white matter at the same time that it increased the sensitivity of the cancer cells to IR [220]. BMX-001 can potentially interact with numerous redox-sensitive pathways, such as those involving NF-κB and Nrf2, thus having an impact on their transcriptional activity [219]. The ability of BMX-001 to reduce the toxic effects of radiotherapy in cancer patients is being evaluated in phase II clinical trials (www.clinicaltrials.gov, accessed on 3 February 2022), e.g., NCT05254327 (rectal Cancer), NCT03608020 (brain metastases), NCT02655601 (high-grade glioma) and NCT02990468 (head and neck cancer) [54], and initial results seem to indicate that BMX-001 reduces side effects of radiotherapy.

#### 6.1.5. Cyclic Nitroxides

Synthetic cyclic stable nitroxide radicals (NRs), such as Tempo, Tempol, XJB-5-131, TK649.030, JRS527.084 or JP4-039, contain a nitroxyl group with an unpaired electron (-NO) and are stabilized by methyl groups, which prevent radical-radical dismutation. In vivo, NRs undergo a very rapid, one-electron reaction to the corresponding hydroxylamine, which has also antioxidant activity. NRs stabilize free radicals, easily diffuse through the cell membranes, have SOD and CAT-like activity, prevent the Fenton and Haber–Weiss reactions and are capable of protecting cells from radical induced damage [54,221].

Gramicidin *S*-nitroxide JP4-039 is a free radical scavenger and antioxidant targeting mitochondria through a segment of a cyclopeptide gramicidin that abrogates mitochondrial oxidative stress and cardiolipin oxidation, playing a pivotal role in the execution of apoptosis. JP4-039 effectively protects and mitigates TBI-induced hematopoietic, GI syndrome and skin damage even when it is delivered intravenously up to 72 h after exposure [222,223]. JP4-039 treatment ameliorated head and neck radiation-induced mucositis and marrow suppression in mice [224]. In a comparative study with other four nitroxides, JP4-039 demonstrated the best median survival after radiation exposition [225]. Based on these properties, Luo et al. have synthesized and analyzed a series of nitronyl nitroxide radical spin-labeled RES derivatives that have also shown important radioprotective effects [226].

#### 6.1.6. Melatonin

*N*-acetyl-5-methoxytryptamine (melatonin), the main secretory product of the pineal gland, is a free radical scavenger with strong antioxidant properties, related to its chemical structure (specifically, the aromatic ring indole rich in delocalized electrons). Melatonin indirectly affects the oxidative–antioxidant balance, stimulating the expression of genes encoding for SODs, GPxs and GR, and ameliorates inflammatory responses. Such protection is evidenced by the capacity of melatonin to reduce 8-hydroxy-2′-deoxyguanosine levels and associated DNA lesions [227,228]. Moreover, animal studies confirmed that melatonin is able to alleviate radiation-induced cell death via inhibiting proapoptotic genes (e.g., Bax) and upregulating antiapoptotic genes (e.g., Bcl-2) [229]. Its radioprotective efficacy in pre-clinical models has been recently reviewed in [230]. Melatonin has some characteristics of an ideal radioprotector (multiple ways of action, low toxicity, and ability to cross biological barriers), and also has anti-cancer properties, i.e., apoptotic, antiangiogenic, antiproliferative, and metastasis-inhibitory effects reviewed in [231]. A meta-analysis of eight randomized controlled trials concluded that melatonin (20 mg, orally administered, once a day) led to substantial improvements regarding tumor remission, 1-year survival, and alleviation of therapy-related side effects [232].

### 6.2. Radiomitigators

Radiomitigators minimize the toxicity of IR even when they are administered after radiation exposure, which differentiates them from radioprotectors that almost prevent/reduce the direct damages. Since most radiological and atomic mishaps are unexpected events, decision-making specialists should consider the use of radiomitigators that can most assist with limiting the destructive impacts of radiation exposure in those already affected. In this technical sense, ideal radiomitigators ought to be anti-inflammatory, enhance antioxidant defenses, have antimutagenic properties, upregulate the DNA repair mechanisms, activate mitotic processes, cell growth and differentiation to promote the regeneration of damaged tissues, and forestall or reduce ARS and CRS. At present, no molecule under study meets all these prerequisites, but there are a large number of choices [54,233,234], which may be combined, for quick administration to affected individuals. For such situations, we may recommend the following:

#### 6.2.1. Antiemetic Drugs, Probiotics, Prebiotics, and Toll-like Receptor Agonists

The pathophysiology of radioinduced GI toxicity is mediated by enterocyte loss, vascular injury, and bacterial translocation. The symptoms involve nausea, vomiting and diarrhea that aggravate electrolyte and fluid loss and lead to morbidity/mortality. Anti-emetics are useful for the stabilization of affected patients, with 5-hydroxytryptamine-3 receptor antagonists (granisetron and ondansetron) often being the first choice of treatment, whereas the addition of dexamethasone provides a modest improvement in prophylaxis [235]. Higher half-life and effectivity make granisetron a better option. The disadvantage of the preventive antiemetic treatment is that prodromal symptoms will be masked and they are useful bioindicators of ARS [235,236].

Gut microbiota dysbiosis aggravates radiation enteritis, reduces the absorbing surface of intestinal epithelial cells, weakens the intestinal epithelial barrier function, and promotes inflammatory factor expression, thus leading to a persistent mucositis, diarrhea and bacteremia [237]. Cancer patients exposed to radiation therapy exhibit marked alterations in gut microbiota composition, with a decrease in protecting *Bifidobacterium* and *Lactobacillus* spp. together with an excessive growth of Gram-negative pathogen bacilli [238]. Maintenance of normal microbiota using probiotics exerts nutrient competition and avoids binding of intestinal pathogens to host mucosa, thus preventing bacterial translocation. Gut microbiota produces short-chain fatty acids (SCFAs), mainly composed of acetate, propionate and butyrate, that are the main energy source of colon cells and prevent intestinal inflammation by reducing the production of chemokines or adhesion molecules. Butyrate, in particular, is reported to stimulate a variety of colonic mucosal functions and to induce the expansion of Treg lymphocytes [239]. SCFAs play an important role in relieving intestinal injury induced by radiotherapy, whereas propionate [240] and valeric acid [241] have shown long-term radiomitigation of hematopoietic and GI syndromes by reducing the release of ROS, DNA damage and proinflammatory responses.

Prebiotics, fecal microbiota transplantation and, especially, probiotics prevent and improve radiation-induced enteritis [242,243]. In preclinical and clinical studies, probiotic interventions with *Lactobacilli* and/or *Bifidobacteria* ameliorate micro-intestinal atrophy and diarrheal symptoms [244], and exert cancer protection [245]. Commensal bacteria and probiotics interaction with Toll-like receptors (TLRs) activate the NF-κB, ensuring the development of innate immune responses, maintaining the barrier function, and promoting wound repair and tissue regeneration [237]. Several TLR2 and TLR4 agonists reduce radiation-induced apoptosis in epithelial stem cells, alleviating intestinal damage [246,247]. In clinical trials, probiotics reduce the incidence of diarrhea [242,243,248] and mucositis in cancer patients treated with radiotherapy [238], even though results are difficult to evaluate as they vary with the type of cancer, radiotherapy modality used, and type of probiotic used [246]. A recently published systematic review concludes that *Bifidobacterium longum*, *Lactobacillus acidophilus*, *Bifidobacterium breve*, *Bifidobacterium infantis* and *Saccharomyces boulardii* could be a good combination to prevent mucositis or ameliorate side effects of radiotherapy [249].

β-glucans (constituents of the cell wall in bacteria and plants) administered prior to and after irradiation exposition, prevent intestinal pathogen bacterial translocation, stimulate hematopoiesis and enhance survival in radiation-exposed animals [233,250]. Urolithin A (UroA), a metabolite generated from the transformation of ellagitannins by the gut, shows immunomodulatory and anti-inflammatory activities, and markedly upregulated the survival of irradiated mice. UroA improved the intestine’s morphology architecture and the regeneration of enterocytes, and significantly decreased radiation-induced p53-mediated apoptotic cell death [251].

#### 6.2.2. Cytokines and Growth Factors

Any radiation dose >2 Gy results in bone marrow depletion, decreased blood cell counts, hemorrhage, and immunosuppression, leading to secondary infections. In the absence of treatment, death may occur in 2–8 weeks post-irradiation. Clinical therapy can help, and should not be limited to the use of antibiotics, blood, and platelet transfusions [236].

Cytokines like IL-1, IL-6, or TNFα promote inflammation, recruit leukocytes into damaged tissues and have restorative effects on the bone marrow. For that reason, earlier studies considered them as radioprotectors [252,253]. Nowadays, this hypothesis has changed since the proinflammatory states exacerbate IR toxicity.

The bone marrow recovery has been highlighted by the FDA, and in fact, some radioprotectants have been approved act in this sense, i.e., Filgrastim (a recombinant DNA type of the physiological G-CSF), Pegfilgrastim (a PEGlylated type of the previous), Sargramostim (a recombinant granulocyte-macrophage colony-stimulating factor, GM-CSF) and recently (2021) romiplostim (a Fc-peptide fusion protein that activates the thrombopoietin receptor) [54,234,254]. G-CSF and pegylated G-CSF promote proliferation, differentiation and maturation, and enhance blood neutrophil recovery and the survival rate. In 2009, The World Health Organization convened a panel of experts to develop recommendations for MCM in the management of H-ARS in a hypothetical scenario involving the hospitalization of 100–200 patients exposed to IR. According to this First Global Consensus, WHO strongly endorsed cytokine therapy (G-CSF or GM-CSF) within 24 h of exposure, above 2 Gy, for affected individuals with significant lymphopenia or when neutropenia (<500 cells/mm^3^) persists for more than 7 days [236,255]. Pegylated G-CSF can be used as an alternative to G-CSF, with the advantage that it can be administered weekly (daily in the case of G-CSF), but it appears to be less efficacious in treating injuries combined with skin burns. Treatment should be maintained until the neutrophil count maintains over 1000 cells/mm^3^ in the absence of infection. Individuals with prolonged anemia can be treated with erythropoietin to avoid transfusions, considering the option of iron supplementation.

GM-CSF, administered as late as 48 h after radiation exposure, accelerates recovery from neutropenia and thrombocytopenia and decreases infection rates [256]. Lung injury (RILI) is a common complication of thoracic cancer radiotherapy, and currently, it has no effective treatment. GM-CSF reduced the occurrence of both pneumonia and pulmonary fibrosis. Moreover, an analysis of the clinicopathological characteristics of 41 patients, undergoing radiotherapy, evidenced that RILI remission was significantly correlated with GM-CSF treatment [257].

Keratinocyte growth factor (KGF) produced by mesenchymal cells protects and repairs epithelial tissues. KGF promotes the recovery of the mucosa, improves intestinal barrier functions and limits bacterial translocation and subsequent sepsis after irradiation. In clinical studies Palifermin^®^, a human recombinant KFG with analogous activity and higher stability, reduced the incidence, duration and severity of oral mucositis and esophagitis in cancer patients, and stimulated immune recovery following hematopoietic stem cell transplantation [258].

Epidermal growth factor (EGF) promotes epithelial and hematopoietic stem cells regeneration [259]. Bone marrow-derived hematopoietic stem cells (HSCs) express the EGF receptor in response to radiation and, in turn, EGF promotes HSCs regeneration in vivo. Mechanistically, EGF reduced radiation-induced apoptosis through repression of PUMA proapoptotic protein, and EGF receptor signaling was needed for DNA repair and for HSCs regeneration [259,260]. rhNRG-1β is an EGF-like protein that maintained mitochondrial integrity and ATP production in irradiated cardiomyocytes and preserves cardiac function via the ErbB2-ERK-SIRT1 signaling pathway [261]. Cotreatment with G-CSF led to a further increase in survival (20% in controls, 67% in EGF, 86% in EGF+G-CSF) [260].

A decrease in fibroblast growth factor (FGF) blood levels is found after irradiation, and a human recombinant derivative (FGF-P) improved duodenal functions and increased survival in GI-ARS mouse models. After been exposed to IR, FGF-P treated animals showed less hemorrhages and cutaneous ulcerations. FGF-P also holds promise for the treatment of burns, wounds and stem-cell regeneration [262].

It must be pointed out that the increased activity of many of these cytokines can be associated with prolonged ROS and RNS generation, a fact that favors the development of chronic inflammatory problems, and thereby the development of fibrosis and/or carcinogenesis [50]. Moreover, many cancer cells (glioblastoma, lung cancer, etc.) increase expression of EGF and other cytokine receptors, which makes the use of these radioprotectors unfeasible in cancer patients undergoing radiotherapy.

Bleeding due to thrombocytopenia is a common cause of death in ARS patients. Several agents have been assessed, including recombinant human thrombopoietin (TPO) and TPO mimetics like romiplostim (Nplate^®^) and eltrombopag [263]. Unfortunately, alloimmunization was developed after TPO administration, and it is no longer manufactured [264]. Nplate^®^ (injectable) activates the TPO receptor on megakaryocyte precursors promoting cell proliferation and platelet production. It has been clinically assayed successfully for the treatment of thrombocytopenia and is approved by the FDA and European Medicine Agency for the treatment of idiopathic purpura and immune thrombocytopenia [265]. Romiplostim (administered for 3 consecutive days) increases survival to 100% in C57BL/6J mice exposed to a γ-TBI (7 Gy) and, at day 30, blood cells, hematopoietic progenitors and the histological appearance of the intestine were similar to non-irradiated controls [266]. Furthermore, a single dose of Nplate^®^ (30 μg/kg) enhanced the survival to 40% [267]; combined with G-CSF and EPO, it increased survival to 100% (0% survival in controls 30 days after exposure), recovering hematological parameters to the levels of non-irradiated mice [268]. In non-human primates, Nplate^®^ and pegfilgrastim combined treatment had much greater effects on platelet and neutrophil recovery following γ-irradiation compared to single agents alone [269]. HemaMax^®^ (human recombinant IL-12) restored all cell progenitor types in the bone marrow, decreased thrombocytopenia, leukopenia and infection rates and preserved GI functions, induced recovery of body weight and increased survival, when administered 24 h post-TBI (8.0 Gy) to mice and rhesus monkeys [270]. Pegylated IL-11 (Neumega^®^) is FDA approved to treat thrombocytopenia in cancer patients, although it has limited use as a radiomitigator, due to the need to be administered daily. To circumvent this problem, another mono-PEGylated IL-11 analog (BBT-059) was designed and showed higher bioavailability and potency in vivo. In a mouse model, BBT-059 led to multi-lineage hematopoietic reconstitution and appears to increase survival more than PEG-G-CSF and PEG-GM-CSF at high TBI doses [259,271].

HSCs and mesenchymal stem cells (MSCs) have also been proven to be effective in treating ARS in preclinical models. Hematopoietic stem cell therapy is recommended for patients with complete aplasia assessed by bone marrow biopsies [255], but in Chernobyl and other accident scenarios, survival was more likely among individuals that did not received bone marrow transplant [272]. Most recipients died shortly after transplantation due to the rapidly progressing insults to skin, lung and gut, complicated by serious bacterial, fungal and/or viral infections [264]. For that reason, Radiation Emergency Assistance Center/Training Site provides recommendations for the administration of antibiotic and/or other antimicrobial agents [264]. The WHO expert group (2011) recommend “wait and see” for a spontaneous or cytokine induction of hematopoiesis recovery, and to consider the administration of hematopoietic stem cells only after 2–3 weeks, and only in the absence of non-hematopoietic organ failure [255]. This recommendation has not changed as a result of the analysis of more recent studies [264]. Mesenchymal stem cells (MSC) are abundant resources (umbilical cord, bone marrow, blood, adipose tissue, and placental tissue), can differentiate into cells of the mesodermal lineage [273], and have demonstrated capacity to regenerate damaged tissues [274]. Despite this promise, translating the potential into actual clinical practice needs to solve many barriers, including immune-rejection, teratogenesis, and others [275]. A clinical trial is evaluating the efficacy of MSC injections for the treatment of chronic radiotherapy-induced complications (PRISME, NCT02814864) [273].

#### 6.2.3. Inhibitors of the Inflammatory Response

Excess of intracellular ROS, hypoxia and microvascular injury induced early activation of HIF-1α is a powerful stimulator of various pro-fibrotic mediators such as TGF-β, chemokines (e.g., MCP-1 and MIP-1beta), vascular endothelial growth factor (VEGF), and platelet-derived growth factor [276,277]. TGF-β stimulates apoptosis through Smad and Rho/Rock pathways, upregulates enzymes such as NOX2, NOX4, COX-2 and iNOS, inducing oxidative stress and proinflammatory responses that may persist and are associated with vascular damages and fibrosis RIBE [278,279,280]. Consequently, it is not surprising that halofuginone (an inhibitor of the TGF-β signaling pathway) and bevacizumab (an anti-VEGF antibody) have been shown to prevent or reduce radiation-induced fibrosis [281,282], with the additional advantage of inhibiting tumor angiogenesis and consequently tumor growth and metastasis formation. Different phase I/II clinical trials in women with metastatic breast cancer have shown more successful radiotherapy response if combined with a TGF-β inhibitor (LY2157299, NCT02538471). In fact, reduction of the plasma levels of TGF-β is associated with greater efficacy of radiotherapy on different types of cancer [283]. Some inflammatory polyphenols (genistein, curcumin, resveratrol or quercetin) downregulate TGF-β expression or signaling pathways attenuating radio-induced skin, pulmonary and/or myocardial fibrosis [102,112,113,145,277].

Radiation exposure enhances COX and iNOS activity, increasing the production of PGE2 and NO (respectively), both involved in the activation of the inflammatory response [284,285]. NSAIDs assayed as radiomitigators include non-selective COX inhibitors, e.g., acetylsalicylic acid (aspirin), ibuprofen, indomethacin, diclofenac, and flurbiprofen. Aspirin ameliorates radiation-induced kidney and lung damage and reduces post-irradiation chromosomal aberrations [286]. A recent meta-analysis of randomized controlled trials indicates that acetylsalicylic acid reduces the overall risk of recurrence and mortality of colorectal cancer and/or colorectal adenomas, which increases the interest in its possible use as a radioprotector/radiosensitizer [287]. Flurbiprofen showed radioprotection in clinical studies, e.g., delaying the onset of mucositis and reducing its severity after radiotherapy in head and neck cancer patients, although the overall severity or duration of mucositis was not improved [288]. Benzydamine (a prostaglandin synthetase inhibitor) decreased the incidence and severity of oral mucositis associated to radiotherapy exposure [289].

Selective COX-2 inhibitors have the advantage of having less undesirable side effects, whereas promote myelopoiesis, thus avoiding the negative feedback control exerted by PGE2 [285]. Meloxicam alone, and in combination with IB-MECA (an adenosine A3 receptor agonist), has been reported to stimulate endogenous production of G-CSF and hematopoiesis, increasing the survival of mice exposed to lethal doses of radiation [290]. Celecoxib (a selective COX-2 inhibitor) attenuated severe skin reactions after a high single dose of 50 Gy and, in rats, reduced brain injury maintaining the integrity of the BBB and reducing inflammation [291]. In a glioblastoma model, the combined effect of radiation and celecoxib increased tumor cell necrosis, showing a significant reduction in tumor microvascular density and prolonged survival compared to irradiation alone [292]. It should be added that the analgesic effects of COX inhibitors can contribute to the well-being of of people affected by exposure to IR.

The mainstay of treatment in acute radiation pneumonitis consists of the systemic administration of glucocorticoids at high doses, aiming to reduce inflammation and inhibit TNFα-induced nitric oxide-mediated endothelial cell and lymphocyte toxicity. The use of inhaled corticosteroids ensures the highest dose deposition in the airway, thus decreasing side effects and ameliorating pulmonary fibrosis [293]. Nevertheless, systematic prophylactic use of corticosteroids to prevent toxic pulmonary edema is not recommended in China or Germany [294] and there is no evidence of a significant long-term benefit based on the use of corticosteroids.

#### 6.2.4. Statins

These 3-hydroxy-3-methyl-glutaryl-coenzyme A reductase inhibitors are commonly used to treat hypercholesterolemia and atherosclerosis. Statins also possess other biological effects, i.e., improving endothelial function, decreasing oxidative stress and inflammation, and regulating the immune system. Statins lessen the mRNA expression of pro-inflammatory and pro-fibrotic cytokines, accelerate the repair of DNA double-strand breaks and mitigate DNA damage [295]. Simvastatin, in particular, has been shown to mitigate radiation-induced enteric injury [296], to prevent radiation-induced marrow adipogenesis [297], to attenuate radiation-induced salivary gland dysfunction in mice [298], and to reduce cardiac dysfunction and capsular fibrosis [299]. GT3 and simvastin provide synergic protection against radiation-induced lethality, hematopoietic and bone marrow injury compared to the single treatments [196]. Pravastatin [300] and atorvastatin [301] have also shown radiomitigative efficacy.

#### 6.2.5. Angiotensin Axis Modifying Agents

There is some evidence that IR upregulates angiotensin II (AngII) expression in a dose-dependent manner, and AngII can increase ROS production through activation of the NADPH oxidase, upregulating inflammatory and profibrogenic pathways involved in long-term radiation injury [302,303]. Moreover, local synthesis of Ang II has been observed in fibrotic plaques and lung myofibroblasts, whereas apoptosis of alveolar epithelial was completely abrogated by an AngII receptor antagonist or by anti-AngII antibodies [304]. In pre-clinical models ACEi (angiotensin-converting enzyme inhibitors) and AngII antagonists, widely used as antihypertensive agents, have been shown to mitigate nephrotoxicity [305], pneumonitis [306,307] and other hematopoietic radio-inducted toxicities [308]. ACEi increases Ang-(1–7) levels which seems to have radioprotective [309] and antitumoral effects [310]. Several retrospective studies reported that ACEi decreased the risk of radiation pneumonitis in lung cancer patients [311]. In a randomized controlled trial in patients exposed to 14 Gy TBI (9 equal fractions for 3 days) captopril mitigated renal nephropathy—increasing survival but not significantly [312], although a subsequent study by the same authors indicated significant differences in survival were attributable to radiomitigating effects on the respiratory system [313]. Captopril has been shown to be a better mitigator than lisinopril, enalapril, or ramipril [234], but a large prospective study in lung cancer patients treated with captopril and radiotherapy was halted due to insufficient accrual [302]. However, a recent meta-analysis seems to evidence that the use of ACEi decreased the incidence of symptomatic radiation-induced pneumonitis in lung cancer patients, especially in those older than 70 years, while those treated with angiotensin receptor blockers had a slight (non-significant) trend towards developing pneumonitis [314]. In addition, recent studies using ramipril and lorsartan showed reduced neuronal apoptosis, enhanced BBB integrity, and improved cognitive and motor function after TBI [315,316], major side effects of cranial radiotherapy in adult and pediatric cancer survivors.

#### 6.2.6. Molecular Hydrogen (H_2_)

The antioxidant advantages of H_2_ gas include [317,318]: (a) selectively scavenging the deleterious ONOO- and •OH radicals, preserving other important ROS and NIS for normal signaling regulation, (b) stronger reductive activity than other dietetic antioxidants as C or E vitamins, (c) enhanced Nrf2 transcription and SOD, CAT and GPx expression [319] and (d) reduced NADPH oxidase activity [320]. In addition to reducing oxidative stress, H_2_ increases the expression of antiapoptotic proteins (Bcl-xL and Bcl-2) [321], which could be helpful to attenuate damage induced by IR. In addition, H_2_ downregulates the expression of adhesion molecules, reduces the infiltration of neutrophils and macrophages [322], inhibits NF-κB and reduces serum IL-1β, IL-6, and TNF-α levels, which could prevent RIBE and alleviate inflammatory response [323]. Most of these properties have been evidenced in a recent human clinical trial [324].

Hirano et al. [325] have recently published an interesting review on the potential radioprotective effects of H_2_ on cognitive function, testis, lungs, heart, skin, cartilage, GI system, hematopoietic organs and the immune system. A randomized placebo-controlled study showed that consumption of H_2-_supplemented water improved the quality of life of patients treated with radiotherapy for liver tumors [326]. H_2_ mitigated radio-induced bone marrow damage in cancer patients without compromising the anti-tumor effects of radiotherapy according to a retrospective observational study [327]. In vitro and in vivo, H_2_-rich water promoted tritium elimination (Table 1), reducing serum levels and tissue-bound tritium, and attenuated the genetic damage [328]. In addition to its antioxidant, anti-inflammatory and antiapoptotic effects, H_2_ can be easily administered through various routes with little adverse effects and great efficacy [317,318,325]. These results show promising potential for the use of H_2_ as a potential radiomitigator that should be studied in more depth. 

#### 6.2.7. Metformin

Metformin is one of the most commonly used anti-diabetic drugs and has shown potential antioxidant, radioprotective, and anticarcinogenic properties [329,330]. It is a hydrogen-rich agent able to neutralize free radicals, increase GSH, and upregulate the activity of SOD and CAT [331], which all favor the antioxidant cell defense. Metformin stimulates DNA repair via non-homologous end joining or homologous recombination, and nucleotide excision repair [50]. Some studies showed that metformin exhibited a radioprotective effect only when administered to mice after radiation exposure; and others evidenced that it can also be considered a radiomitigator, because it reduced chronic production of ROS and pro-fibrotic cytokines such as TGF-β, and attenuated fibrosis through modulation of pro-oxidant genes such as NOX4, if administered after radiation exposure [332]. Metformin can also induce several redox-related genes, such as the PRKAA2 (which encodes the AMPK), a mechanism that helps in protecting cells from the accumulation of unrepaired DNA and attenuates inflammation and pro-fibrotic pathways [329]. Metformin also ameliorates IR hematopoietic stem cell injury in mice [333]. Cardiovascular disease is a pivotal disorder after radiotherapy and the administration of metformin to γ-irradiated (5 Gy) rats significantly ameliorated the increase in plasma of cardiac disease-related biomarkers such as, LDH and CK-MB, NF-κB, IL-6 and TNF-α levels compared to the control group, which suggests that concomitant administration of metformin during radiotherapy can act as an efficient heart protector from oxidative stress and inflammatory damages, and endothelial dysfunction-derived damage [334]. Furthermore, several studies have evidenced the synergistic action of metformin when it is administered with sulfhydryl containing drugs [335] or with melatonin [332,336], although in others the synergy was not evidenced and melatonin was shown to be a better radioprotector [337]. It is also worth mentioning that metformin improves tumor oxygenation and the response to radiotherapy in tumor xenograft models. Thus, it can be considered a potential radiosensitizer to improve the outcome of radiotherapy [338,339]. In this regard, the use of metformin in patients with hepatocellular carcinoma and receiving radiotherapy has been associated with a higher overall survival [340]. Metformin’s anticancer effects are well documented in preclinical studies, along with early phase clinical trials, but there is a significant lack of late phase clinical trials [341].

As we noted previously, we do not have any molecule that meets the requirements of an ideal radioprotector or radiomitigator. However, a combination of molecules may accomplish summation of protective/mitigating mechanisms and, in the end, synergies. Moreover, it is basic to have MCM that can be effective and that can also be immediately administered to the people in need. Such combinations should be assayed in standard clinical trials. Fortunately, most of the alternatives referenced above have had such preclinical and clinical examinations performed for various indications (see e.g., https://www.clinicaltrials.gov, accessed on 3 February 2022).

### 6.3. Radionuclide Scavengers

Table 1 summarizes the diverse radionuclides that might be encountered in contamination events. Clinical countermeasures and treatments, their route of administration and main mechanisms of action, as well as the organs or tissues where those agents may accumulate are also indicated. Radiation exposure brought about by radionuclide contamination only stops if the radionuclide is completely disposed from the body, with or without therapy.

**Table 1:** Contamination by radionuclides and medical countermeasures.

Ionizing Radiation Type	Element	Radioactive Half-Life	Major Exposure Pathways	Focal Accumulation	Medical Countermeasure for Internal Contamination	Mechanism of Action	Route of Management	References
**α**	**Americium** (^**241**^**Am**)	432.7 years	Inhalation Skin	Lung Liver Bone Bone marrow	Calcium- diethylenetriaminepentaacetate (Ca-DTPA)	Ca-DTPA is 10 times more effective than Zn-DTPA, thus Zn-DTPA is considered a second choice. It is also a good option for long term treatment.	IV (single infusion, do not fractionate the dose). Nebulized inhalation (only in adults) Wound irrigation can be accompanied by IV DTPA. Within the first 24 h. Drink a lot and void frequently.	[11,342,343]
					Zinc- diethylenetriaminepentaacetate (Zn-DTPA)			
	**Plutonium** (^**238**^**Pu**, ^**239**^**Pu** and ^**240**^**Pu**)	88, 24,100 and 6563 years, respectively	Inhalation (Limited absorption)	Lung Bone Bone marrow Liver Gonads	Ca-DTPA/Zn-DTPA	Chelating agent (see above).	IV (daily bolus or single infusion, do not fractionate the dose). Nebulized inhalation (only in adults).	[175,343,344,345]
					Deferoxamine (DFOA) (methanesulphonate of deferoxamine B)	Pu–deferoxamine complex is urinary excreted.	IM (preferred route) IV (slow infusion)	
					Hydroxypyridinonate ligand 3,4,3-LI(1,2-HOPO)	Quelating agent, with increased urinary and predominant biliary Pu excretion.	Oral or IV. Not approved in humans, but it has a wider window of use and is more effective than DTPA.	
	**Polonium** (^**210**^**Po**)	138.4 days	Inhalation Ingestion Skin	Spleen Kidneys Lymph nodes Bone marrow Liver Lung mucosa	Dimercaprol (BAL) (First choice)	^210^Po reacts with thiol groups, so chelating agents containing these (BAL or DMPS) increase its renal excretion.	IM, but do not continue for too long. Repeated doses of BAL in combination with DMSA reduce the retention more than BAL alone.	[173,174,346]
					Dimercaptosuccinic acid (DMSA)	Forms water soluble chelates and, consequently, increases the urinary excretion of ^210^Po.	Oral. Safety/efficacy in children <12 years is not established.	
					Deferoxamine	^210^Po-chelates are excreted in the urine.	IM (preferred route) or IV (slow infusion)	
					D-Penicillamine		Oral, avoid if case of penicillin allergy	
					Dimercaptopropansulphonate (DMPS)		Oral or IV	
					Gastric aspiration or lavage	Reduce intestinal absorption in case of ingestion	It only has some effect within 2 h of intake.	
					Magnesium sulfate, aluminum hydroxide, or barium sulfate		Oral	
	**Thorium** (^**232**^**Th**)	1.41 × 10^10^ years	Inhalation Ingestion	Bone	Ca-DTPA/Zn-DTPA	Chelating agent (see ^241^Am)	(See ^241^Am)	[143]
	**Uranium** (^**235**^**U** and ^**238**^**U**)	7.1 × 10^8^ and 4.5 × 10^9^ years respectively	Inhalation Ingestion	Kidneys Bone	Sodium bicarbonate (NaHCO_3_)	Alkalinization leads to the formation of UO_2_(CO_3_)_3_^4−^ which is quickly eliminated by the urine, preventing toxic nephritis. Chelating agents must not be used.	IV (until urine pH is 8–9, and then mantain for 3 days)	[347,348,349]
				Kidneys	Acetazolamide	Reduces uranium tubular reabsorption accelerating renal excretion. 3 times more effective than NaHCO_3_, but without effect on skeletal retention	Oral	[350]
**β**	**Phosphorus** (^**32**^**P**)	14.3 days	Inhalation Ingestion Skin	Bone Bone marrow Rapidly replicating cells	Aluminum carbonate	Phosphate binder	Oral (for 5 days)	[351]
					Sodium glycerophosphate	Phosphate competition	Oral	
					Sodium phosphate		Oral	
					Potassium phosphate		Oral	
					Potassium phosphate, dibasic		Oral (with a glass of water)	
					Sevelamer carbonate	Phosphate binder	Oral	
					Aluminum hydroxide	Competitive inhibition of intestinal absorption.	Oral	
	**Strontium** (^**89**^**Sr** and ^**90**^**Sr**)	51 days and 28 years, respectively	Inhalation Ingestion	Bone	Stabile strontium	Competitive inhibition of intestinal absorption.	Oral or IV	[352,353,354,355,356]
					Ammonium chloride (First choice)	Acidification of the medium favors the formation of anionic forms and their renal excretion.		
					Calcium gluconate (First choice)	Competes for bone binding sites based on chemical analogy.	IV (within 12 h, if it is possible)	
					Sodium alginate, for digestive contamination.	Used for the treatment of gastro-oesophageal reflux. It forms a high viscosity gel and block intestinal absorption.	Oral (with a full glass of water)	
					Calcium carbonate	Ca interferes with intestinal absorption of Sr and Ra, increases fecal excretion, and compete with their bone deposition	Oral (within 12 h of radionuclide intake if it is possible)	
					Calcium phosphate	Phosphate decreases intestinal absorption, whereas Ca increases urinary excretion of Sr-90.	Oral (single dose within 24 h of radionuclide intake)	
					Barium sulfate	Reduces the intestinal absorption by forming insoluble sulfates.	Oral (single dose within 24 h)	
					Aluminum phosphate	Reduces intestinal absorption.	Oral	
					Aluminum hydroxide	Reduces intestinal absorption.	Oral	
	**Tritium** (^**3**^**H**)	12.5 years	Inhalation Ingestion Skin	Whole-body	Water	Facilitates urinary excretion. The effectiveness of dilution will be greater, the sooner it is applied.	Oral (>3–4 L/day, for 3 weeks). H_2_-rich water accelerates ^3^H renal excretion.	[327,357]
					Chlorthalidone	Diuretic. Increase the renal elimination of ^3^H and other radionuclides.	Oral	
					Furosemide	Diuretic. Increase the renal elimination of ^3^H and other radionuclides	Oral	
	**Yttrium** (^**90**^**Y**)	64 h	Inhalation	Bone	Ca-DTPA/Zn-DTPA	Chelating agent (See ^241^Am)	See ^241^Am, but the administration must be done within 4 h	
					EDTA	Chelates are urinary excreted	IV (single dose) or IM (divided dose)	
**β, γ**	**Cesium** (**^134^Cs and ^137^Cs**)	2 and 30 years, respectively	Inhalation Ingestion	Whole-body (especially kidney)	Prussian Blue (Ferric hexacyanoferrate)	Traps Cs in gut minimizing intestinal absorption and reabsorption (in the case of Cs radionuclides excreted in the bile).	Oral	[358,359,360,361]
	**Cobalt** (**^57^Co, ^58^Co** and **^60^Co**)	272 days, 71 days, and 5.26 years, respectively	Inhalation	Whole-body (especially liver)	EDTA	Chelates are urinary excreted	IV (Single dose) or IM (divided dose)	[362,363,364]
					Dimercaprol	Forms chelates by competing with endogenous sulfhydryl groups.	IM	
					Ca-DTPA/Zn-DTPA	Chelating agent (see ^241^Am)	(See ^241^Am)	
					Cobalt gluconate	Isotopic dilution.	IM or sublingual	
	**Iodine** (**^131^I**)	8.1 days	Inhalation Ingestion Skin	Thyroid	Potassium iodide (KI)	Competitive inhibition of NIS reduces thyroid deposition. Iodide blocks 98% of I-131 thyroid uptake if it is administered several minutes before incorporation.	Oral. Single dose that must be administered less than 24 h prior to, and up to 2 h after exposure.	[343,359,365]
					Potassium perchlorate (KClO_4_)	Competitive inhibition of NIS reduces thyroid deposition. Not recommended in pregnant and newborns	Oral. Alternative option for iodine sensitivity. In Japanese is more effective than IK.	[365,366]
	**Iridium** (**^192^Ir**)	74 days	N/A	Spleen	EDTA	Chelating agent (see cobalt).	IV (single dose) IM (divided dose)	[367,368]
					Ca-DTPA/Zn-DTPA	Chelating agent (see ^241^Am)	(See ^241^Am)	
**α, γ**	**Californium** (**^252^Cf**)	2.6 years	Inhalation Ingestion	Bone Liver	Ca-DTPA/Zn-DTPA	Chelating agent (see ^241^Am)	(See ^241^Am)	[369]
**α, γ, neutron**	**Curium** (**^244^Cm**)	18 years	Inhalation Ingestion	Liver Bone	Ca-DTPA/Zn-DTPA	Chelating agent (see ^241^Am)	(See ^241^Am)	[175,343,369,370]
**α, β, γ**	**Radium** (**^226^Ra**)	1602 years	Ingestion	Bone	Aluminum hydroxide	Blocks intestinal absorption.	Oral	
					Calcium gluconate	Competes for bone binding sites based on chemical analogy.	IV (within 12 h, if it is possible)	[355,371,372,373]
					Calcium carbonate	(See above in ^90^Sr)	Oral	
					Calcium phosphate	(See above in ^90^Sr)	Oral	
					Sodium alginate	(See above in ^90^Sr)	Oral (with a full glass of water)	
					Barium sulfate	Reduces the intestinal absorption of ^226^Ra by forming insoluble sulfates	Oral	

MCM as listed have been suggested as treatments for internal contamination with radioisotopes by: Hickman, D. P. Management of persons contaminated with radionuclides: NCRP Report No. 161 (Volume 1) [374]. *Medical Management of Persons Internally Contaminated with Radionuclides in a Nuclear or Radiological Emergency*; Emergency Preparedness and Response; International Atomic Energy Agency: Vienna, 2018 [375]. Hübner, K.F.; Watson, E.E. Management of Persons Accidentally Contaminated with Radionuclides: NCRP Report No. 65. Washington, D.C. 1980 [376]. Managing Internal Radiation Contamination—Radiation Emergency Medical Management Available online: https://remm.hhs.gov/int_contamination.htm (accessed on 5 May 2022) [377]. Ammerich, M.; Giraud, J.M.; Helfer, N.; Menetrier, F.; Schoulz, D.; Blanc, J.; Vilain, D.; Boll, H.; Bourguignon, M.; Chappe, P.; et al. Medical Intervention in Case of a Nuclear or Radiological Event—National Guide, Release V36, 2008 [378].

### 6.4. Biological Dosimetry

Despite the fact that there are many approved and expected biomarkers to survey the harmful impacts of IR [54], which biomarkers ought to be suggested in the instance of a radiological or atomic crisis? In these scenarios, speed, reliability, and traceability should be the prevailing criteria. These needs rouse us to choose, as the most suitable, those presented below.

#### 6.4.1. Lymphocyte Depletion Kinetic (LDK) Assay

This measure is utilized to gauge the dose following whole or incomplete body outer radiation exposure which was absorbed over minutes to hours. Methodologically, serial complete blood counts are acquired, and the outright lymphocyte check is determined and followed over time. The typical reach for outright lymphocyte count can be impacted by numerous variables, including the hardware utilized, and the ethnicity, age, health, and sex of the examined reference population. In addition, lymphocyte counts can be decreased or expanded by medications, contamination, and numerous clinical problems disconnected from radiation. What is key is that the lymphocyte exhaustion rate is directly identified with radiation assimilated dose (dose range 0.5–14 Gy). For example, a dose of 2–4 Gy associates with lymphocyte atrophy happening over ~4–6 days, while for a dose of 4–6 Gy the lymphocyte decrease requires ~2–4 days [379]. The US Armed Forces Radiobiology Research Institute (AFRRI) BAT (biodosimetry assessment tool) program (https://www.remm.nlm.gov, accessed on 15 February 2022) proposes acquiring a blood cell count as soon as possible after radiation exposure, and suggests that if the absorbed dose is known or suspected to be ≥5 Gy, blood counts ought to be obtained each 9–12 h for 2 to 3 days after irradiation and afterward at regular intervals of 24 h for 3 to 9 days. Other than this, a >5 Gy gamma-ray equivalent dose can be estimated based on biological end points like initial vomiting <2 h after exposure, or other dosimetric end points like physical dosimetry. If the retained dose is known or suspected to be <5 Gy, blood counts ought to be acquired at regular intervals of 24 h for 9 days. A straightforward dose-prediction algorithm based on lymphocyte kinetics as documented in prior radiation accidents was proposed by Goans et al. [380], where results are determined in gamma dose (Gy) whole body counterparts. Notwithstanding, this technique has impediments since it is not appropriate for surveying fractional body exposures or internally deposited radioisotopes.

In developed nations confronting a large-scale radiation crisis, biodosimetry dependent on LDK, clinical signs and indications, and dose estimated from geographic data are probably going to be accessible more quickly than biodosimetry dependent on cytogenetics [31].

#### 6.4.2. Neutrophils-to-Lymphocytes (NLR) Ratio

The neutrophil-to-lymphocyte ratio (NLR) is a valuable marker of host inflammation, which mirrors the connection between circulating neutrophils and lymphocyte counts (dose rage 0.5–10 Gy). It may conveniently be determined from routine complete blood counts (CBCs) with differentiation. It has been indicated that an increase in NLR throughout radiotherapy has a negative impact on survival in breast cancer patients, putting these patients with radiotherapy-susceptible host immunity at a higher risk of tumor recurrence [381]. An essential investigation of the prognostic estimation of the NLR compared with human whole-body irradiation was published after the mishap at the Chernobyl Nuclear Power Station [382].

#### 6.4.3. Cytogenetics

Cytogenetic dosimetry is a significant dose evaluation strategy, especially when there are challenges in deciphering the information, in scenarios where there is reason to believe that people not wearing dosimeters have been exposed to radiation, in instances of cases claiming for compensation after suffering radiation harms that are not upheld by unequivocal dosimetric proof, or in instances of exposure over a person’s working lifetime (https://www.iaea.org, accessed on 24 February 2022). This incorporates [383,384] the examination of:

1. DNA lesions. IR can cause a wide range of harm in DNA including base damage (BD), single-strand breaks (SSB), abasic sites (AS), DNA-protein cross-links (DPC), and DNA twofold strand breaks (DSB).
2. Chromosome-type abnormalities, i.e., dicentrics (DC, the worldwide standard for cytogenetic biodosimetry after radiation exposure; the dicentric yield is utilized to assess the radiation exposure measurements as per a measurably inferred and predetermined dose-reaction plot) [385]; centric rings; acentrics; rogue cells; reciprocal, non-reciprocal and interstitial translocations (dose range 0.5–5 Gy).
3. Chromatid-type aberrations i.e., terminal and interstitial deletions; achromatic injuries; isochromatic deletions; asymmetrical interchanges; symmetrical interchanges; triradials.
4. Premature chromosome condensation (PCC). The G0-PCC can be a valuable instrument for high dose biodosimetry with a speedy evaluation of fragment recurrence (dose range 0.2–20 Gy). Further, it holds the potential for multi-parametric dosimetry in combination with fluorescent in situ hybridization (FISH) [386].
5. Micronuclei framed from slacking chromosomal fragments or whole chromosomes at anaphase which are excluded from the nuclei of daughter cells (dose range 0.2–5 Gy). The cytokinesis-blocked micronucleus test (CBMN) is a biodosimetric technique to gauge chromosomal harm in mitogen-stimulated human lymphocytes [387]. While this procedure produces dose-reaction information that fits a straight quadratic model for exposures to low LET radiation and dosages up for 5 Gy, restrictions to the precision of this technique emerge at bigger doses. Precision at higher dosages is restricted by the number of cells at mitosis.

In this way, thinking about the technical conditions around a radiological or atomic crisis, it appears reasonable to suggest DSB, DC, and CBMN tests as the most ideal choices. For example, Nakamura et al. [388] considered the causal connection between DNA harm acceptance in bovine lymphocytes and the Fukushima mishap. DNA harm was assessed by evaluating the degrees of DNA DSB in peripheral blood lymphocytes by immunocytofluorescence-based measurement of γ-H2AX foci (dose range 0.5–5 Gy (microscopy) or 0.5–10 Gy (cytometry)). A more than two-fold increment in the fraction of harmed lymphocytes was seen in all animal cohorts within the evacuation zone. These outcomes set up a clear relationship between exposure and elevated levels of harm to DNA in animals living in the area of the atomic power plant mishap.

#### 6.4.4. Other Options

Other likely biomarkers of IR-induced harm incorporate (but are not restricted to) oxidative stress markers (e.g., 8-hydroxy-2′-deoxyguanosine, isoprostanes and protein carbonyls), immune and inflammatory mediators (various cytokines and chemokines), altered gene expression and mutations (e.g., NF-κB activation, GADD45, CDKN1A, genes related with the nucleotide excision repair mechanism, TP53, PPP1R14C, TNFAIP8L1, DNAJC1, PRTFDC1, KLF10, TNFAIP8L1, Slfn4, Itgb5, Smim3, Tmem40, Litaf, Gp1bb, Cxx1c, FDXR), epigenetic markers (gene methylation and repetitive components), metabolomics-related markers (e.g., urine glyoxylate, threonate, thymine, uracil, citrate,2-oxoglutarate, thymidine, 2′-deoxyuridine, 2′-deoxyxanthosine; blood serum inositol, serine, lysine, glycine, threonine, glycerol, isocitrate, gluconic acid, stearic acid, methylglutarylcarnitine), proteomics-related markers (e.g., plasma ferredoxin reductase, α-2-macroglobulin, chromogranin-A, GPx-3, lipidomics-related markers (e.g., blood serum linoleic acid, palmitic acid, phosphatidylcholines, glycerolipids, glycerophospholipids and esterified sterols), and miRNAs (e.g., miR-150, miR-30a, miR-30c, miR-34a, miR-200b, miR-29a, miR-29b, miR-144-5p, miR-144-3p). As of now, the hardware required, the need for automation (see for example [54]), and the absence of explicit investigations of this topic, do not prompt us to recommend any of these choices as satisfactory in a radiological or nuclear mishap scenario.

#### 6.4.5. Networks

The WHO set up in 1987 the REMPAN organization (Radiation Emergency Medical Preparedness and Assistance Network) in light of the tasks assigned to it in the conventions on early notice and help with the event of atomic mishaps, for which the International Atomic Energy Agency (IAEA, https://www.iaea.org, accessed on 1 March 2022) is the responsible association. In 2007, WHO directed an overview of biological dosimetry laboratories and their crisis reaction abilities in chosen districts. The outcomes demonstrated a robust capacity, although there were not many local or public organizations set up. WHO BioDoseNet was then implemented as a worldwide organization of biodosimetry laboratories whose job is to help in management and decision-making in instances of big radiation crisis events where the capacity of individual labs is likely to be overwhelmed. Global biodosimetry networks have been set up, such as the Latin American Biological Dosimetry Network (LBDNet), the organization from Canada and The United States of America (North American BD Network), the Chromosome Network Council coordinated by Japan, the Asian Network of Biological Dosimetry (ARADOS), the Biological Dose Network in China and the European Network for Biological and Retrospective Physical Dosimetry (RENEB). At global level, worldwide organizations have been set up by the WHO (BioDoseNet), the IAEA (within RANET), EURADOS, and the Global Health Security Initiative (GHSI). This speaks to coordinated worldwide action to organise dose assessment. As an illustration of systems management efficacy, the MULTIBIODOSE project (multi-disciplinary biodosimetric instruments to oversee high scale radiological victims), established as a feature of the FP7 Euratom program in May 2010, showed genuinely similar outcomes among various research centers, with reliable dose estimates [389].

Systems management should give: (1) agility, by offering permanent support, every day/365 days a year, (2) prompt admittance to a facilitated server with stand-up capacity for integration, and (3) use of same equipment and supplies, standard operating procedures (SOPs), alignment curves and scoring rules for validated tests. Well built-up coordination can give the upgraded capacity to react to either demand for help from entities without dose assessment capability, or those who may be overwhelmed due to an abrupt surge of patients with suspected or known exposures. Consequently, in the event that one research center gets overwhelmed, tests can be shipped off to different labs within the network with certainty that the dose assessments will be reliable and equivalent.

#### 6.4.6. Advances in Automation

An ideal reaction to radiological or nuclear crises (large-scale specifically) suggests the need for multi-parametric examination combined with a quality assurance/control, speed in collecting samples, high-throughput technology for test planning and analysis, right linkage with clinical triage and treatment surge, and proficient data management frameworks.

The US Biomedical Advanced Research and Development Authority (BARDA) program (https://www.phe.gov, accessed on 7 March 2022) is currently financing two correlative classifications of biodosimetry innovations: (1) A point of care test for a brisk triage of an exposed populace, categorized as having a biological dose above or under 2-Gy to determine the danger of suffering ARS. This test is intended to be managed by an individual with next to zero clinical preparation in a field hospital or triage station. Preferably, results would be accessible in less than 15 min after getting the sample with minimal sample handling. The point of this test is to isolate the people with radiation-related clinical necessities from the individuals who may not need explicit therapy. (2) A lab based high-throughput assay to quantitatively evaluate the dose retained by a person. The framework being created is fit for evaluating the assimilated dose in the range of 0.5–10 Gy with a superior exactness contrasted with the point-of-care devices [390]. This framework is required to be capable of processing up to 400,000 samples per week with a high level of lab computerization. Automation in obtaining results after sample assortment would in a perfect world be under 8 h. Over time, the utilization of robotized platforms and the improvement of research facility surge capacity networks can help customary cytogenetic evaluation techniques. In this sense, enhancements incorporate the utilization of barcoded test compartments, mechanical fluid handlers, and computerized metaphase cell collectors, metaphase cell spreaders, slide stainers, and coverslippers [385]. Two primary methodologies have been utilized to diminish the time expected to gauge a dose—first, the work of robotized metaphase finders, and second, decreases in the number of metaphases scored. A few programming platforms have been created for triage management utilizing existing biodosimetry procedures; for example, time to emesis, LDK, and DCA (dicentric chromosome assay) have been implemented for use in the point-of-care setting. The BAT program (see above) is a model. The DCA QuickScan strategy further speeds up scoring [391]. Additionally, the RABIT-II-DCA is a completely computerized DCA in multiwell plates. All activities, from test stacking to chromosome scoring, are performed, without human mediation, by the second generation Rapid Automated Biodosimetry Tool II (RABiT-II) mechanical framework, a plate imager, and custom programming, FluorQuantDic. The framework requires small volumes of blood (30 µL per individual) to appraise the radiation dose received because of a radiation mishap or terrorist assault [392].

The CBMN is a biodosimetric instrument to measure chromosomal harm in mitogen-stimulated human lymphocytes. A scanning and image processing system with a robotized micronucleus scoring, the Radometer MN-Series (RS-MN) microscopic system designed by Radosys (Budapest, Hungary, https://www.radosys.com, accessed on 15 March 2022), has been presented for triage [393]. A similar model is the CytoRadx Assay (https://asell.com, accessed on 15 March 2022).

The FAST-DOSE (Fluorescent Automated Screening Tool for Dosimetry) is an immunofluorescent, biomarker-based framework intended to reproduce assimilated radiation dose in blood tests from possibly exposed people. This framework is intended to evaluate intracellular protein changes in blood leukocytes, and has been shown to effectively differentiate beneath or over 2 Gy as long as 8 days after complete body exposure in non-human primates [394].

The G0-PCC (G0-Phase Premature Chromosome Condensation) permits chromosome aberration analysis within the space of hours after blood assortment. Among all deviations, the examination of chromosomal fragments is the fastest [386]. Significantly, this approach holds potential for multi-parametric dosimetry in combination with FISH.

Mechanization of sample analysis by flow cytometry may overcome the time restrictions connected to the utilization of magnifying instrument-based examination (e.g., γ-H2AX identification [395]). This a phosphorylated type of the H2A histone family member X forming when twofold strand breaks show up, and it has been proposed for screening radiation-initiated DNA harm [396].

The DosiKit is a field-radiation biodosimetry immunoassay for fast triage of people exposed to outer TBI, which was validated in human blood cell extracts 0.5 h after in vitro exposure to ^137^Cs γ rays, utilizing γ-H2AX analysis. DosiKit can appraise absolute body irradiation doses from 0.5 to 10 Gy with a solid linear dose-dependent signal and can be utilized to differentiate possibly exposed people into three dose ranges: under 2 Gy, in the range of 2 to 5 Gy, and above 5 Gy (DCA permits exact estimation of dosages under 5 Gy). The fundamental preferred position is a brisk test that can be performed directly in the field by operational personnel with minimal preparation [397]. The DosiKit framework was completely integrated into a deployable radiological crisis research lab, and the reaction to operational necessities was exceptionally favorable [398].

The REDI-Dx Biodosimetry Test System (https://www.redidx.com, accessed on 15 March 2022) has been created as an in vitro analytic test, which uses blood collected into DxCollect^®^ Blood Collection Tubes (BCT) for the quantitative assessment of the absorbed IR dose. Test outcomes are analyzed with the ABI 3500xL Dx Genetic Analyzer (Rancho Dominguez, CA, USA) and the REDI-Dx Interpretive Analysis Software (Rancho Dominguez, CA, USA), for the gene expression of a set of radiation responsive genes based on the DxDirect^®^ genomic platform. REDI-Dx has been demonstrated to be a good indicator of dosage, for deciding treatment classification dependent on either 2.0 or 6.0 Gy [399].

The HemoDose is a software device, which estimates absorbed doses based on blood counts (https://www.remm.nlm.gov, accessed on 21 March 2022) [400]. The dose assessed by HemoDose dependent on lymphocyte counts and DC chromosome indicated an equivalent correlation with hematological ARS degrees 1 and 4 (in light of the clinical therapy protocols for radiation mishap casualties, METREPOL) [401,402].

### 6.5. Biophysical Dosimetry

#### 6.5.1. External Exposure

##### Personal Dosimetry

These devices should be utilized when individuals are in danger of exposure to IR. The chest or stomach locations are predominant. Dosimeters can be additionally positioned in a limb for the scenario where the assessed dose received could be higher in the limb than in the rest of the body [403]. A standard individual dosimeter ought to be fit for giving data on ingested dosages from photons of at least 10 Gy (https://www.iaea.org, accessed on 21 March 2022). Naito et al. [404] estimated individual external doses during the recovery phase in a town after the Fukushima Daiichi atomic power plant mishap. They utilized an individual dosimeter (D-Shuttle) combined with a global positioning system gadget to quantify, and subsequently comprehend, individual outer doses relying upon the resident’s area. At the point when a mishap happens outside the controlled territory, exposed people not wearing dosimeters cannot be checked for radiation exposure.

##### Area Monitoring

This is not expected to survey individual doses, but to give a rough estimate of the dose rate at the mishap site [405]. The requirement for exact and fair-minded radiation monitoring is exemplified by the disarray with respect to radiation levels around the Fukushima nuclear site [406]. Examinations may include different likely areas and hotspots for the evaluation of the presence and power of radioactivity in the environment and for the affected people. Region monitors ought to work dependent on explicit measures for the required level of precision, considering the reliance on radiation energy, direction of incidence, temperature, radiofrequency interference, as well as other expected factors.

Instruments used in screening a territory can include: (a) instruments for photons; (b) instruments to detect β particles and low energy photons; (c) instruments for neutrons; (d) passive γ monitors; (e) passive neutron survey meters; and (f) spectrometers (https://www.iaea.org, accessed on 21 March 2022). For example, after the Fukushima fiasco, most information with respect to the diminishing of biological radioactivity was estimated utilizing a NaI (Tl) scintillation meter (Hitachi Aloca Medical, Ltd., TCS-172B), aligned according to the International Electrotechnical Commission’s norms (IEC 60846-1:2009) [407].

##### Dose Reconstruction

Dose reproduction is a review assessment of radiation dose(s) received by recognizable or representative people from a specific exposure. Much of the time, it is the only strategy to assess γ radiation or low dose exposure. Numerous factors, e.g., distance from the source, exposure length, irradiation geometry, and shielding, should be considered in dose assurance, making this technique a tedious system (https://www.icrp.org, accessed on 21 March 2022).

As depicted by [408], scientific issues in radiation dose recreation can be assembled into three distinct classifications: (a) information issues, for example, demographic data, changes in site tasks over the long run, characterization of intermittent versus persistent exposures, and the utilization of colleagues’ information; (b) dosimetry issues, for example, strategies for evaluation of exposures, missed dose, unmonitored dose, and clinical radiation dose brought about as a condition of the workplace; (c) explicit issues identified with outer dose, such as affectability, precision and energy reliance of individual monitors, exposure geometries, and ongoing uncertainties. Issues identified with inner dose incorporate sensitivity of bioassay techniques, uncertainties in biokinetic models, suitable dose coefficients, and modelling uncertainties.

As instances of this strategy, Ivanova et al. [409] built up a system for the reproduction of individualized exposure doses for people dwelling at Chernobyl at the hour of the mishap. The strategy depends on the information of radio-biological (ground, meal) and dosimetric (whole body estimations) checking held in Ukraine during the period 1986–2013. Related to the Fukushima atomic mishap, Technical Report No. 162 (2012) gives a thorough record of the estimations and studies undertaken by the Australian Radiation Protection and Nuclear Health Agency (https://www.arpansa.gov.au, accessed on 1 April 2022) to survey the effect on the health of people and the environment in Australia. This report incorporates radiation observations of the environment and seas and testing of imported food and merchandise.

##### Electron Paramagnetic Resonance (EPR)

EPR dosimetry depends on the evaluation by EPR spectroscopy of dose subordinate changes in the concentration of free radicals, defects, or any species with paramagnetic properties that is shaped in a given material under exposure to IR [410]. The capacity of electron paramagnetic reverberation to quantify radiation-derived paramagnetic species, which persist in specific tissues (e.g., teeth, fingernails, toenails, bone, and hair), has made this procedure a noteworthy technique for screening significantly exposed people [411] for dose range 1–30 Gy.

##### Optically Stimulated Luminescence (OSL)

Luminescence signals utilized in dosimetry comprise light emitted under stimulation by a material able to store energy from radiation. Such materials incorporate insulators and semi-conductors [410]. The standard method for estimating OSL is to illuminate the sample with a steady power incitement source and measure the resulting luminiscence (identified as CW-OSL). The OSL signal arrives at the greatest outflow very quickly after the irradiation is turned on, and from that point decays dramatically as the snares are discharged [412]. The essential points of interest when contrasted with EPR are that there is no spectral deconvolution required and the equipment needed is considered less complex and more suited to field events. The applicable dose range is 0.03–10 Gy.

##### Thermoluminescent (TL) Material

TL-detectors were used for cosmic radiation dosimetry in early 1960s, and since then they have been applied in numerous space missions for personal dosimetry, for biological experiments and for medical applications [413].

The TL material has the capacity of storing energy when presented to IR. This energy is re-discharged as visible light when the material is heated to a suitable temperature. The unadulterated materials with ideal grid structure are not considered as TL materials; however, when certain materials are added (which serve as activators), they display thermoluminescence [414]. TL materials can demonstrate the environment radiation dose at the site of a mishap but not the assimilated dose of a victim.

Dose computation utilizing luminescence of solid-state dosimeter has become a significant field of innovative work, and has been effectively applied in territories affected by the Hiroshima and Nagasaki bombs [415], at the Nevada test site [416], and the Semipalatinsk test site [417]. The strategy was additionally utilized for areas affected by the Chernobyl mishap [418] and in contaminated settlements of the upper Techa River in the Southern Urals [419]. The applicable dose range is 0.01 mSv–10 Sv.

#### 6.5.2. Internal Exposure

##### Whole-Body Counters (WBC)

A WBC is a gadget to measure principally γ-rays emitted by radioactive material present in the body, which may vary contingent upon the radionuclide. Alpha particle decaysa can likewise be identified by their coincident γ radiation [420]. Detection can be accomplished by utilizing either a scintillation indicator or a semiconductor locator set in proximity to the body. A fundamental constraint of this strategy is that WBC might not be able to distinguish radioisotopes that have comparable γ energies.

##### Thyroid Monitoring

After the Chernobyl mishap, thyroid malignancy expanded, a problem that was particularly evident among children who had internal exposure to radioiodine through milk consumption [365,421]. This was expected due to the inclination of radioactive iodine to collect in the thyroid and children’s thyroids having a higher susceptability to radiation than adults. For that reason, radiological prophylaxis must be dedicated primarily to ensuring that the measure reaches, in optimal conditions, children and young people under 18 years of age, pregnant and lactating women [365].

^131^I has a short half-life of around 8 days; however, once it enters the body, a high percentage will accumulate in the thyroid, and the gland will thus be directly exposed to β-particles and γ-beams. A reduction of radioiodine uptake into the thyroid can be achieved by administering a large dose of IK (130 mg, in adolescents older than 12 years and adults) shortly before and up to 2 h after, the expected onset of radiation exposure [365,366]. Saturation of the sodium/iodide symporter (NIS) and the Wolff–Chaikoff effect are the main mechanisms involved in transient blockage of ^131^I uptake in the thyroid [422]. As indicated in Table 1, stable iodine blocks about 98% of ^131^I thyroid uptake if it is given several minutes before incorporation. If the administration is simultaneous, the efficacy drops to 90%, being of the order of 50% when the iodine is administered 4–6 h later. Administering IK 24 h after exposure can even be counterproductive, because it prolongs the biological half-life of ^131^I that is already accumulated in the thyroid [365]. A single administration of IK is normally enough, although repetitive administrations might be required in the case of prolonged or repeated exposures. The latter is not recommended in neonates orpregnant and breastfeeding women due to the risk of adverse effects [365,366].

NIS transports other monovalent anions, with the following decreasing activity: TcO_4_^−^ > ClO_4_^−^ > I^−^ > Br^−^. Perchlorate has a higher affinity to the NIS than iodide, thus it can be a good alternative in case of iodine sensitivity [365,366]. The Japanese have a delayed responsiveness to iodine transport saturation; thus, potassium perchlorate confers a much better protection in acute ^131^I exposure. In case of longer or repeated exposures, preference should be given to perchlorate in both Caucasians and Japanese [366].

##### Lung Monitoring

Lung checking is desirable not long after the intake, as it gives a more precise estimation of lung deposition and retention than whole-body estimation [423]. Suggestions have been made for computation of radiation dosages to the respiratory tract of laborers exposed to airborne radionuclides (Human Respiratory Tract Model for Radiological Protection, https://www.icrp.org, accessed on 1 April 2022). The retention is given for the complete body (for example, activity in all compartments of the biokinetic model, including respiratory tract and the thoracic lymph nodes). These capacities are determined several times after an acute intake (for example, inhalation or ingestion).

Lung counting is the sort of in vivo estimation suggested for radionuclides with long residence times in the lung, for example, uranium oxides, plutonium, and ^241^Am oxides. In vivo estimations of radionuclides in the lung commonly include the detection of X rays as well as photons with energies < 200 keV (https://www.iaea.org, accessed on 1 April 2022).

##### Bioassays

Concerning contamination, a bioassay is characterized as the assurance of amounts of radioactive material in the human body, regardless of whether by direct estimation, in vivo checking, or by measuring materials discharged or eliminated from the human body (for example the utilization of nasal swipes and swabs to evaluate for inhalation; stools and urine to survey for ingestion; and excisional biopsies of cutaneous scraped spots, slashes, and soft tissue wounds to evaluate for transdermal retention or absorption through injuries) [424].

After the Fukushima mishap in 2011, it was decided at the National Radiation Triage Medical Center (NREMC) of the Korea Institute of Radiological and Medical Sciences that mobile units for inward contamination checking would be more effective than the utilization of fixed-type WBCs to screen individuals. Accordingly, the NREMC developed a Mobile Radiobioassay Laboratory (MRL) for fast field-based checking of internal contamination following atomic or radiological crises [425].

#### 6.5.3. Body Surface Contamination

In the early phases of an atomic mishap, it is important to screen the surface radioactive contamination of everyone living and working around the accident site (https://www.irpa.net, accessed on 1 April 2022). Proper survey meters ought to be utilized. In such a situation, radiation type, size of contaminated territory, and strength and compactness of the detector itself are key determinants. Surface contamination is estimated by surface review meters, for example, a Geiger–Müller (G-M) counter, which is valuable for the concentration level, adequacy of the disinfecting method, and skin dose [426]. During evacuation following the Fukushima mishap, evacuees were screened for body surface contamination utilizing a G-M meter [201]. Body surface contamination levels should be related to inhaled thyroid dosages.

#### 6.5.4. Neutron Exposure

The neutron activation strategy is based on the measurement of radiation released by the decay of radioactive nuclei formed by neutron irradiation of the samples (body organs or tissues). At the point when radioactive atoms decay in the sample, γ rays with traceable energies are emitted by each nuclide. Utilizing a γ-ray spectroscope, the amount and energy of radiated γ rays can be estimated. For a mishap associated with neutron emission, neutron activation is the best technique to assessing the dose [204]. Ekendahl et al. [427] assessed neutron exposure to radioactivity in body tissues utilizing samples of human blood and hair dependent on neutron-spectrum calculations.

## 7. Conclusions

**Figure 1:**
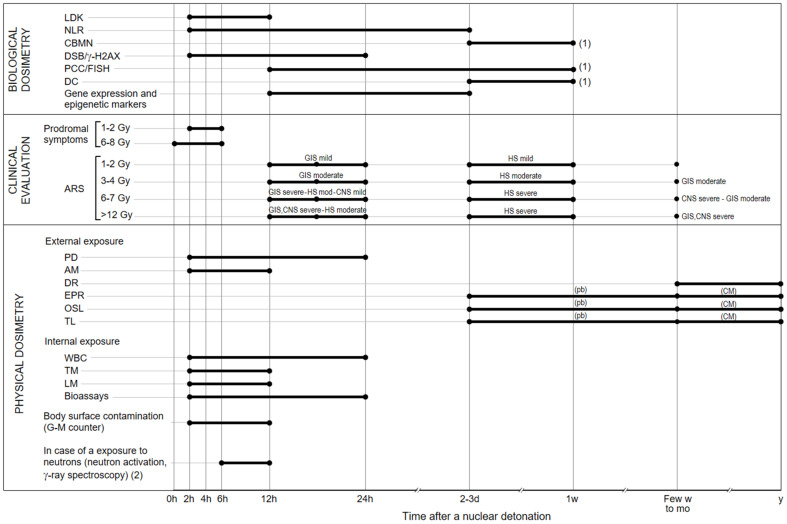
Stepwise dosimetry and evaluation after a nuclear detonation. (1) Results can be obtained more quickly if automation systems are available. GIS, gastrointestinal syndrome; HS, hematopoietic syndrome; CNS, central nervous system syndrome; mod, moderate; (pb), personal belongings; CM, construction materials. (2) Samples of human blood and hair. These cytogenetic techniques require 48–72 h to process the samples (not indicated). We have focused on the specific case of samples which are processed right after the accident, in a scenario where triage is rapid to avoid deterministic effects. If there is no option, samples can be processed later, and the results used in a retrospective manner. A recognized drawback of the dicentric (DC) and cytokinesis-block micronucleus (CBMN) assays is that the damage is unstable and therefore can be eliminated from the peripheral blood lymphocyte pool. Nevertheless, some lymphocytes containing aberrations continue to exist in the peripheral circulation for many years after an irradiation, although a high dose exposure or a long delay between irradiation and sampling can reduce the aberration yield. Sevan’kaev et al. remark that “the pattern of decline was biphasic with a more rapid first phase, with a half-life of 4 months, followed by a slower decline with half-lives around 2–4 years. It is usually assumed that for biological dosimetry purposes, where delayed sampling requires an extrapolation to zero time, the yield of DIC decreases with a half-life of about 3 years” [428]. Therefore, DIC and CBMN are useful for dose assessment from 2–3 days to 3 years after the accident, whereas FISH, as a stable alteration, has a time window of more years. It is recommended to run these assays within the first year after harmful radiation exposure.

Biological and biophysical dosimetry is fundamental in distinguishing the individuals who need prompt clinical mediation from those with a possibility for postponed therapy, those who only require long follow up, and those potentially requiring no medical care. However, as of now, biomarkers and procedures do not appropriately fit a triage scenario. As no single biodosimetry method is adequate for dose prediction, time points, and exposure conditions, use of a combination of various methodologies is essential. Biomarker research likewise faces constraints, notably, on experimentation with humans, albeit sometimes the clinical exposure to therapeutic radiation has created informative outcomes. Moreover, most investigations have been performed utilizing a single type of radiation. Nonetheless, e.g., photon and neutron dosages in mixed exposure scenarios ought to be evaluated independently, since this may be key to assessing the danger of radiation-induced clinical syndromes. Lab information is basic to help decision-making, yet biomarker data are not sufficient for a health evaluation, triage, therapy, and clinical management. Research on biomarkers for dose assessment ought to incorporate exposure to mixed field radiation (synchronous exposure to various sorts of radiation), internal exposures from inhalation or ingestion of radionuclides, accumulated serial exposures, and combined injury (as the inflammatory reaction will occur in organs/tissues affected by injuries, which will confound the assessment). Finally, the utilization of radiation biomarkers to anticipate levels of exposure should address the characteristic differences in radiosensitivities across a population. As a supplement to the biodosimetry procedures, we additionally recommend the option of utilizing biobanks to safeguard samples that do not need prompt examination. Consequently, a multiparametric approach based on physical, biological, and clinical strategies appears the most proper decision. For instance, Figure 1 lays outs the dosimetry/assessment measures that, with the available technology, ought to be performed in the event of a nuclear explosion.

During an accidental radiation exposure, MCM should separate first responders and individuals directly exposed to radiation. Ideal radioprotectors or radiomitigators for such scenarios have not been found. Based on recent advances and studied mechanisms, in this review we have discussed those that in our opinion appear most promising. Radioprotectors include derivatives of aminothiols and cyclic nitroxides (which, despite having toxic effects, have been shown to have greater radioprotective efficacy), natural products (vitamin antioxidants, trace elements such as Se, phytochemicals, etc.), and antioxidant enzyme mimetics, among others. Much more has been achieved in terms of increasing the knowledge about the mechanisms related to RIBE and in reducing side effects of radiotherapy in cancer patients. These advances will help in the development of new radiomitigating strategies. Among the most promising therapies are those aimed at activating recovery of the tissues (cytokines and growth factors), preventing side effects (probiotics, prebiotics, etc.), reducing the inflammatory response (bevacizumab, COXi, angiotensin axis modifying agents, statins) and, thereby, radio-induced chronic side effects such as fibrosis or others. The combination of antioxidant and anti-inflammatory effects of many of them prevents DNA damage and reduces the risk of developing tumors and/or cancers. Some molecules (e.g., melatonin, metformin, curcumin, caffeic acid, bevacizumab, etc.) offer the additional advantage of increasing the efficacy of radiotherapy on cancer cells, and thus can be used as radiosensitizers in cancer cells. Given that none of the tested molecules have total radioprotective/radiomitigating effects, it is evident that more work is needed to implement combined strategies aiming to find synergistic and/or additive effects. The development of new formulations (nanoparticles, nanocrystals, nanovesicles) will facilitate oral administration or the release of said molecules in especially sensitive tissues, thereby contributing to effective radioprotective/mitigating doses and reducing possible toxic effects. In the event of a nuclear emergency, the protocols and time sequence for selecting/caring for the affected exposed, as well as the appropriate treatments (radioprotectants/radiomitigators), including doses and route of administration, need to be implemented. MCM are still assigned FDA orphan drug status, and thus many of these radioprotective strategies could be considered by FDA under “fast track” approval process.

The utilization of complementary tools, preferably shaping parts of automated equipment and established networks, is the future of radioprotection research. Furthermore, in practice, clinical evaluation faces restrictions for large scale screening, i.e., the need of uniquely prepared medical care workers or the low throughput due to the short time available to finish a correct evaluation. Straightforward strategies such as an early lymphocyte count are needed to set up a baseline. However, this may not be conceivable in a large-scale event because of time limitations and the absence of enough technical personnel.

Therefore, despite the many advances discussed in this review, there are many challenges that still need to be addressed to deal effectively with nuclear and radiological accidents.

## Abbreviations

AA, acid ascorbic; ACEi, angiotensin-converting enzyme inhibitors; AFRRI, United States armed powers radiobiology research institute; AngII, angiotensine II; AP sites, apyrimidinic sites; ARADOS, Asian network of biological dosimetry; ARS, acute radiation syndrome; AS, abasic sites; ASA; acetylsalicylic acid; BARDA, United States biomedical advanced research and development authority; BAT, biodosimetry assessment tool; BBB, Blood-Brain Barrier; BBT-059, PEGylated interleukin-11; BCT, Blood Collection Tubes; BD, base damage; CA, caffeic acid; CAPE, caffeic acid phenethyl ester; CAT, catalase; CBCs, complete blood counts; CBMN, cytokinesis-blocked micronucleus assay; COX, cyclooxygenase; CNS, central nervous system syndrome; CRS, chronic radiation syndrome; DC, dicentric chromosome; DPC, DNA-protein cross-links; DSB, double strand breaks; EGCG, Epigallocatechin-3-gallate; EGF, epidermal growth factor; EPR, electron paramagnetic resonance; FAST-DOSE, fluorescent automated screening tool for dosimetry; FDA, Food and Drug Administration; FGF, fibroblast growth factor; FISH, fluorescent in situ hybridization; G-CSF, granulocyte colony-stimulating factor; GHSI, global health security initiative; GI, gastrointestinal; GI-ARS, gastrointestinal ARS; GIS, gastrointestinal syndrome; GM-CSF, granulocyte macrophage colony-stimulating factor; G-m, Geiger-Müller; GPx, Glutathione peroxidase; GSH, L-γ-glutamyl-L-cysteinyl-glycine; GSSG, oxidized glutathione; GT3, γ-tocotrienol; H-ARS, hematopoietic acute radiation syndrome; HIF-1α, hypoxia-inducible factor-1α; IAEA, international atomic energy agency; IEC, international electrotechnical commission; IND, improvised nuclear devices; iNOS, inducible nitric oxide synthase; IR, ionizing radiation; JP4-039, Gramicidin *S*-derived nitroxide; KGF, Keratinocyte growth factor; LBDnet, latin American biological dosimetry network; LDK, lymphocyte depletion kinetic assay; LET, linear energy transfer; LSS, Life Span Study; MCM, medical countermeasures; Melatonin, *N*-acetyl-5-methoxytryptamine; MRL, mobile radiobioassay laboratory; NAC, *N*-acetylcysteine; NF-κB, nuclear factor kappa-light-chain-enhancer of activated B cells; NIS, sodium/iodide symporter; NREMC, national radiation Triage medical center; Nrf2, Nuclear factor erythroid 2-related factor 2; NRL, neutrophils-to-lymphocytes ratio; OSL, optically stimulated luminescence; PCC, premature chromosome condensation; POC, point of care; PPARs, peroxisome proliferator-activated receptors; RABIT-II, rapid automated biodosimetry tool II; RED, radiation exposure devices; RDD, radiological dispersal devices; REMPAN, radiation Triage medical preparedness and assistance; RENEB, European network for biological and retrospective physical dosimetry; RES, resveratrol; RILI, Radiation-induced lung injury; RNS, reactive nitrogen species; ROS, reactive oxygen species; RS-MN, radiometer MN-series; SALT, Sort Assess Lifesaving Interventions Treatment/Transport; SCFAs, short-chain fatty acids; SOD, superoxide dismutase; SOP, standard operating procedure; SSB, single strand breaks; TNF-α, tumor necrosis factor alpha; TGF-β, transforming growth factor beta; TBI, total body irradiation; TL, thermoluminiscent material; TLRs, Toll-like receptors; TM, thrombomodulin; UroA, urolithin A; VEGF, vascular endothelial growth factor; WBC, whole body counters.
